# Alogliptin improves survival and health of mice on a high‐fat diet

**DOI:** 10.1111/acel.12883

**Published:** 2019-01-15

**Authors:** Biao Zhu, Yixiang Li, Lingwei Xiang, Jiajia Zhang, Li Wang, Bei Guo, Minglu Liang, Long Chen, Lin Xiang, Jing Dong, Min Liu, Wen Mei, Huan Li, Guangda Xiang

**Affiliations:** ^1^ Department of Endocrinology Wuhan General Hospital of Chinese People's Liberation Army Wuhan China; ^2^ Department of Hematology and Medical Oncology, School of Medicine Emory University Atlanta Georgia; ^3^ Allconnect Inc. Atlanta Georgia; ^4^ Clinical Center of Human Gene Research, Union Hospital, Tongji Medical College, Huazhong University of Science and Technology Wuhan China

**Keywords:** DPP‐4 inhibitor, GLP‐1, healthspan, high‐fat diet, longevity

## Abstract

Alogliptin is a commonly prescribed drug treating patients with type 2 diabetes. Here, we show that long‐term intervention with alogliptin (0.03% w/w in diet) improves survival and health of mice on a high‐fat diet. Alogliptin intervention takes beneficial effects associated with longevity, including increased insulin sensitivity, attenuated functionality decline, decreased organ pathology, preserved mitochondrial function, and reduced oxidative stress. Autophagy activation is proposed as an underlying mechanism of these beneficial effects. We conclude that alogliptin intervention could be considered as a potential strategy for extending lifespan and healthspan in obesity and overweight.

## 1 INTRODUCTION

The number of overweight and obese individuals worldwide has increased dramatically over the past 30 years, leading to an explosion of obesity‐related health problems associated with increased morbidity and mortality. Patients with obesity exhibit many manifestations of accelerated aging, such as cardiovascular disease, diabetes, hypertension, dyslipidemia, nonalcoholic fatty liver disease and inflammatory disorders, all of which reduce lifespan. For these reasons, ways to improve the obesity‐related health problems for extending longevity are actively pursued.

Autophagy is an evolutionarily conserved intracellular catabolic pathway in which damaged organelles and macromolecules are degraded while amino acids are recycled through lysosome, which is stimulated during periods of starvation (Mizushima, Levine, Cuervo, & Klionsky, 2008) or inhibited in long‐term high‐fat diet (Park & Lee, 2014). With aging, the ability of autophagic degradation declines and consequently causes accumulation of damage leading to a broad spectrum of age‐associated dysfunctions (Cuervo, 2008). Whereas autophagy activation supposedly restores cellular function during aging by enhancing degradation of damaged cellular components and that probably is a central process in extending longevity (Johnson, Rabinovitch, & Kaeberlein, 2013). Thus, a reagent served as an activator of autophagy might provide beneficial effects on promoting lifespan.

Dipeptidyl peptidase 4 (DPP‐4) inhibitors and glucagon‐like peptide‐1 (GLP‐1) analogues improve glucose metabolism through activation of GLP‐1 receptor signaling, which induce insulin secretion and suppress glucagon secretion in the pancreas (Drucker & Nauck, 2006). Apart from glycemic actions, lots of animal studies suggest the beneficial effects of DPP‐4 inhibitors on age‐related diseases, such as improvement of insulin resistance and delay of the onset of diabetes (Kim et al., 2012), inhibition of obesity‐related inflammation (Zhuge et al., 2016), suppression of atherosclerotic lesion formation (Matsubara et al., 2012), and reduction of reactive oxygen species (ROS; Femia et al., 2013). Meanwhile, some clinical studies have also shown the beneficial effects of DPP‐4 inhibitors on age‐related diseases, such as improvement of vascular endothelial function (Poppel, Netea, Smits, & Tack, 2011), and protection of the heart from ischemic left ventricular dysfunction (McCormick et al., 2014) in type 2 diabetes. Furthermore, accumulating data demonstrate that GLP‐1 has beneficial effects on age‐related diseases in animal and human (Alvarez‐Villalobos, Trevino‐Alvarez, & Gonzalez‐Gonzalez, 2016; Marso et al., 2016; Nikolaidis et al., 2004; Ye et al., 2010). Of note, the LEADER study (Alvarez‐Villalobos et al., 2016) has found that liraglutide (a GLP‐1 analogue) treatment was associated with reduced primary outcome (by 12.8%), cardiovascular mortality (by 21.7%), and all‐cause mortality (by 14.5%) in type 2 diabetes. Importantly, recent studies have revealed that DPP‐4 inhibitors, GLP‐1 and GLP‐1 analogues induced cytoprotective autophagy in vivo and in vitro (He, Sha, Sun, Zhang, & Dong, 2016; Liu, Liu, & Yu, 2016; Zhou, Zhang, & Zhu, 2014).

The broad pleiotropic effects on attenuating multiple age‐related diseases of DPP‐4 inhibitors and GLP‐1 analogues raise the possibility that they may take beneficial effects on extending lifespan. Therefore, we hypothesized that DPP‐4 inhibitors play a potential role in extending lifespan. In this study, we explored the potential effects of alogliptin, a highly selective DPP‐4 inhibitor, on survival and health of mice on a long‐term high‐fat diet.

## 2 RESULTS

### 2.1 Increased lifespan

To select optimum dose of alogliptin, we firstly performed the dose–response experiment in apolipoprotein E null (ApoE^−/−^) mice on high‐fat diet. Naturally, ApoE^−/−^ mice exhibited shorter lifespan compared with C57BL/6 mice (Chirico et al., 2016; Honma et al., 2013). Cohorts of one‐year‐old male ApoE^−/−^ mice were provided with high‐fat diet (60% of calories from fat, HFD) for the remainder of their lives. To the diet, we added the drug in two doses, consisting of 0.01% or 0.03% (wt/wt) alogliptin estimated from previous studies (Moritoh, Takeuchi, Asakawa, Kataoka, & Odaka, 2008; Shah et al., 2011) and found the higher dose was more prominent on increasing survival. Thus, we selected 0.03% (wt/wt) alogliptin as the intervention dose (Supporting Information Figure s1a).

**Figure 1:**
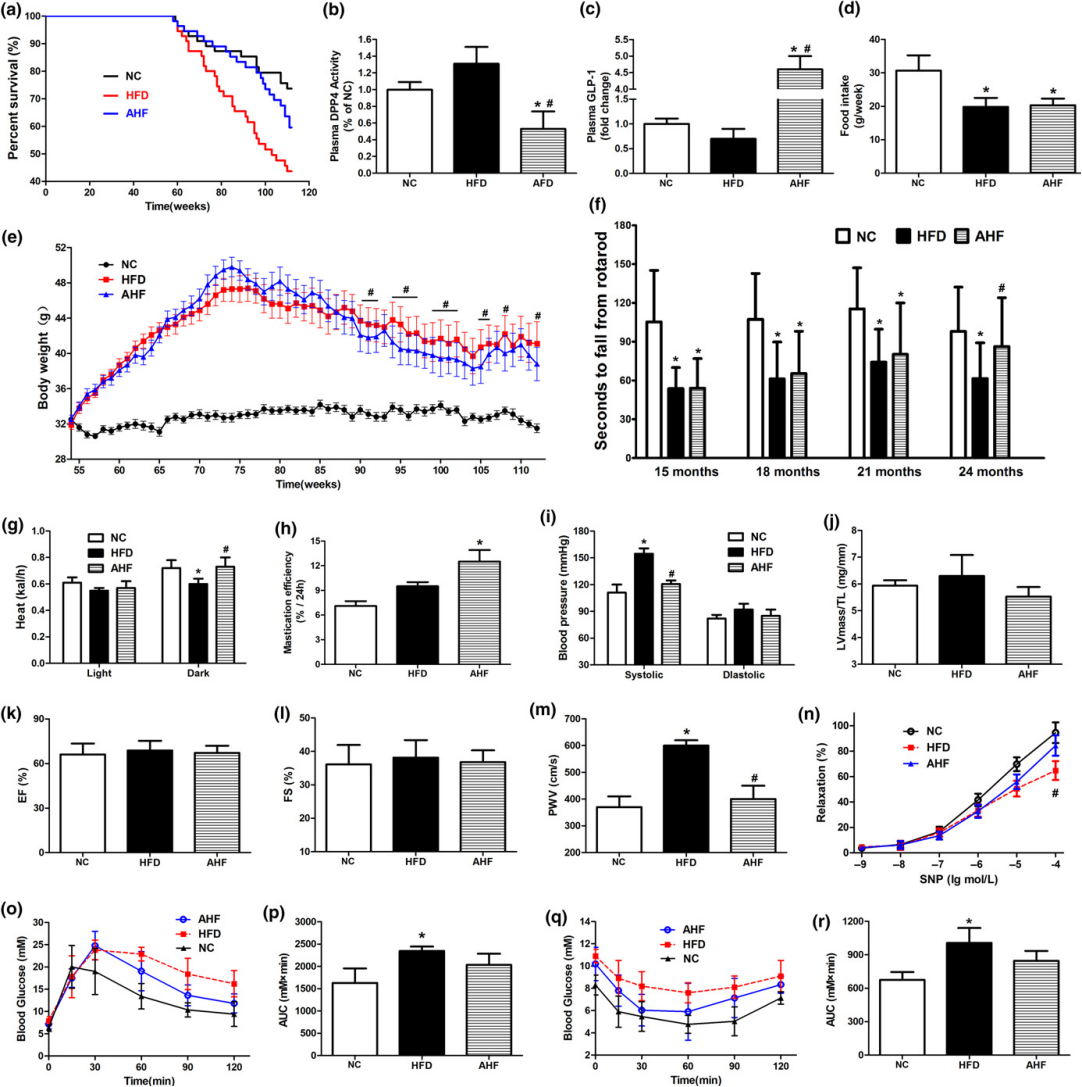
Alogliptin intervention increases survival and improves physical performance. (a) Kaplan–Meier survival curves. Fifty‐five mice for each group. (b) DPP‐4 activity and (c) GLP‐1 protein level in plasma. (d) Food intake. (e) Body weight. Data are represented as mean ± 0.5 *SD*. (f) Time to fall from an accelerating rotarod. (g) Energy expenditure. (h) Mastication efficiency. (i) Blood pressure. (j) Ratio of left ventricular mass to tibia length (LV mass/TL). (k) Ejection fraction (EF). (l) Fractional shortening (FS). (m) PWV. (n) Endothelium‐independent vasodilation function of aortas in response to sodium nitroprusside (SNP). (o) IPGTT. (p) Area under IPGTT curve. (q) ITT. (r) Area under ITT curve. NC, normal chow. HFD, high‐fat diet. AHF, 0.03% (wt/wt) alogliptin plus HFD. Data are expressed as mean ± *SD* except where noted. *p < 0.05 compared with NC, ^#^ *p < *0.05 compared with HFD

In this dose, the effects of alogliptin intervention on plasma DPP‐4 activity and GLP‐1 levels were examined in 13 months old mice. Interestingly, plasma DPP‐4 activity trended to increase in the HFD group compared with NC (normal chow) group (*p* > 0.05), while it was significantly inhibited in alogliptin plus HFD (AHF) group when compared with the other two groups (Figure 1b), and consequently preserved a relatively higher level of GLP‐1 which decreased in HFD group (Figure 1c).

At about 64 weeks of age, the survival curves of HFD and AHF groups began to separate. With the present age at 112 weeks, 60.0% of mice in HFD group had died (median survival 103 weeks), compared to 45.5% in AHF group and 31.0% in NC group (Figure 1a). Although we could not predict the ultimate mean lifespan extension, comparison of survival curves showed that alogliptin intervention markedly increased lifespan compared to HFD group in the log‐rank survival test (*χ* ^2^ = 3.950, *p* = 0.047), but not significantly different from NC group (*χ* ^2^ = 1.882, *p* = 0.170). In addition, sitagliptin, another DPP‐4 inhibitor, took a similar longevity‐promoting effect (*χ* ^2^ = 4.231, *p* = 0.040; Supporting Information Figure S1b), although the structural similarity between sitagliptin and alogliptin is only 31.3% (Wang et al., 2016).

The mean weight of HFD mice steadily gained until 76 weeks of age, after which slowly declined (Figure 1e). From 90 to 107 weeks of age, AHF mice were lighter than HFD mice, consistent with a previous study (Shah et al., 2011) showing alogliptin intervention significantly reduced body weight. But no differences were found in food intake (Figure 1d), total feces mass, or lipid content in the feces (Supporting Information Figure S1c,d). Next, we examined whether the decreased body weight was due to higher energy expenditure. Metabolic experiments showed heat production of AHF group was significantly higher than HFD group in dark phase (Figure 1g).

### 2.2 Attenuated functional decline and improved health

Although lifespan was extended, it was also important to ascertain whether health was improved. Therefore, we performed a series of experiments to measure physiological functions of several vital organs in the old mice.

Firstly, rotarod was performed to test motor coordination, and results indicated that alogliptin intervention improved balance ability of the laboratory mice in AHF group. At 24 months of age, they were even indistinguishable from NC group (Figure 1f). Interestingly, alogliptin intervention also elevated mastication efficiency in AHF mice (Figure 1h); secondly, high‐fat diet elevated systemic blood pressures, especially systolic pressure, and these were attenuated by alogliptin intervention (Figure 1i). Thirdly, it is established that cardiac dysfunction is a significant predictor of death in mice and humans (Dai et al., 2009; Eisenberg et al., 2016). Tibia length‐normalized left ventricular mass (LV mass/TL) is an indicator of cardiac hypertrophy, and ejection fraction (EF) and fractional shortening (FS) are properties of cardiac systolic function. The echocardiography results showed that these parameters were all less affected by alogliptin intervention (Figure 1j–l; Supporting Information Figure S2); Next, stiffening of large elastic arteries is a strong and independent risk factor of cardiovascular events on senescence (Fleenor et al., 2014; Mitchell et al., 2010). We found alogliptin intervention reduced pulse wave velocity (PWV) on high‐fat diet to level similar to NC mice (Figure 1m). We also examined endothelium‐independent vasodilation function of aortas in response to sodium nitroprusside (SNP). Results showed that long‐term high‐fat diet decreased the vascular vasodilation function, which was remarkably improved by alogliptin intervention (Figure 1n); finally, intraperitoneal glucose tolerance test (IPGTT) and insulin tolerance test (ITT) displayed that HFD mice had significantly lower glucose disposal ability than NC mice, while alogliptin intervention exhibited an improvement in glucose and insulin tolerance (Figure 1o–r).

### 2.3 Decreased organ pathology

Our above animal experiments showed alogliptin intervention improved physiological functions on high‐fat diet, and we next investigated whether the structures of organs were also benefited.

**Figure 2:**
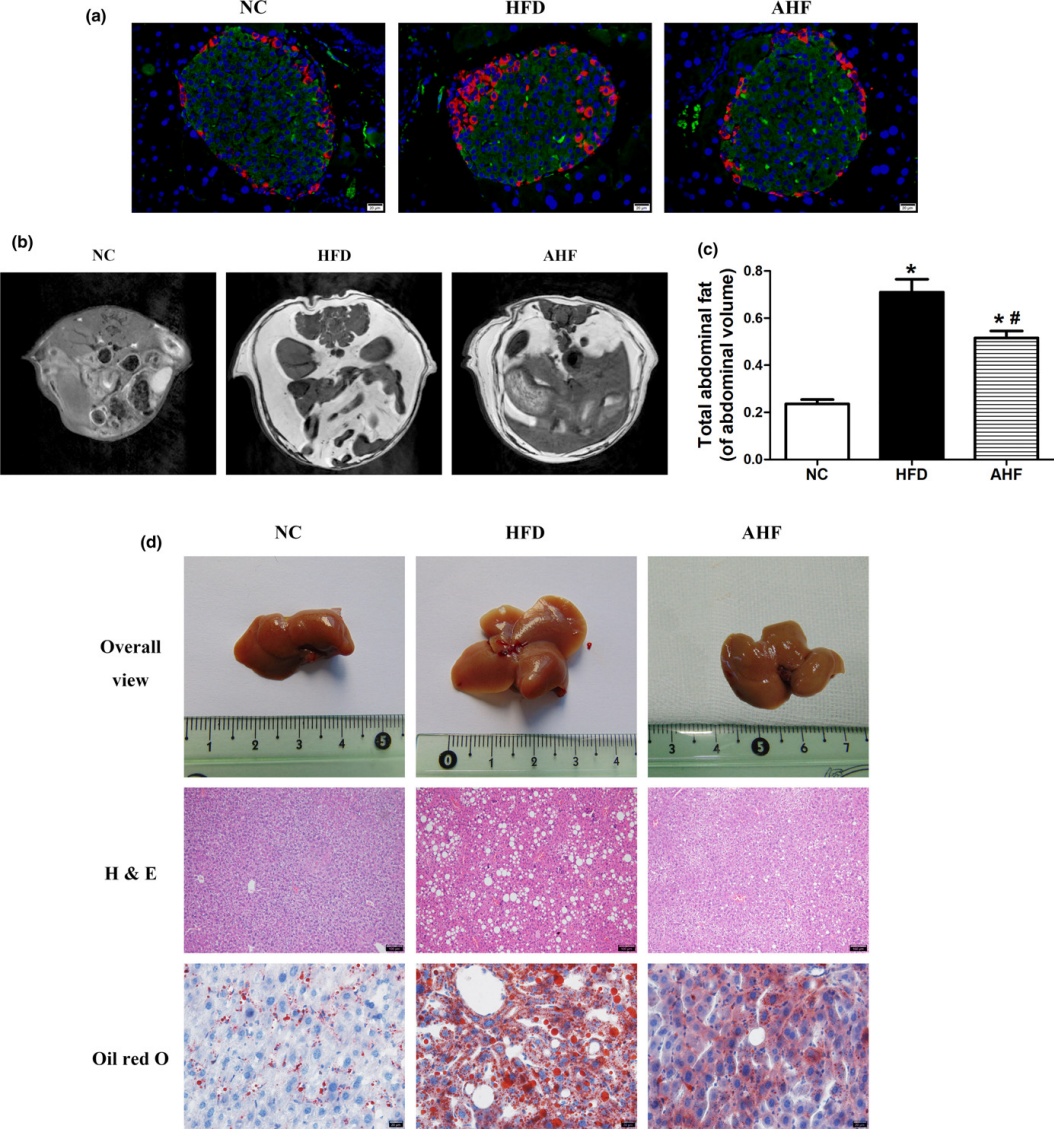
Alogliptin intervention improves organ pathology of pancreas and livers. (a) Representative images of pancreatic sections double‐stained for insulin (green) and glucagon (red), the nuclei were stained with DAPI (blue). Scale bar, 20 µm. (b)T1‐weighted spin‐echo images and (c) analysis of fat mass distribution. (d) Alogliptin intervention prevents the development of fatty liver as assessed by organ size and decreases fat accumulation as measured by hematoxylin‐eosin (H&E) staining (scale bar, 100 µm) and oil red O staining (scale bar, 20 µm). NC, normal chow. HFD, high‐fat diet. AHF, 0.03% (wt/wt) alogliptin plus HFD. Data are expressed as mean ± *SD*. *p < 0.05 compared with NC, ^#^ *p < *0.05 compared with HFD

Long‐term high‐fat diet deteriorates insulin sensitivity leading to diabetes and decreases longevity (Baur et al., 2006), and *β*‐cell dysfunction progresses to a reduction in mass is one of pathological fundaments in diabetes (Takeda et al., 2012). In our study, high‐fat diet increased glucose and insulin in blood, and alogliptin intervention attenuated these increases (Table 1). Indeed, histological examination of pancreas showed islets from AHF mice exhibited a large insulin‐positive cell core encompassed with an orb of α‐cells, paralleling NC mice, while the islet architecture of HFD mice was disorganized (Figure 2a). Measurement of islet β‐cell proportion revealed a 16.34% decrease on high‐fat diet (*p* < 0.05), and which was normalized with alogliptin intervention (Supporting Information Figure S3a).

**Table 1:** Effects of high‐fat diet and alogliptin on blood biomarkers

Parameter	Normal chow (NC)	High‐fat diet (HFD)	Alogliptin + HFD (AHF)
Glucose‐fasted (mM)	6.3 ± 0.9	7.9 ± 2.1*	7.1 ± 1.7
Glucose‐fed (mM)	9.5 ± 1.2	10.9 ± 2.5	10.2 ± 2.2
Insulin (mIU)	13.45 ± 1.84	29.81 ± 2.87*	15.52 ± 2.01#
IGF‐1 (ng/ml)	367 ± 45	402 ± 37	411 ± 33
HbA1c (%)	5.3 ± 0.7	5.7 ± 0.6	5.2 ± 0.9
TG (mM)	1.19 ± 0.15	2.45 ± 0.19*	1.98 ± 0.22* ^,^ #
TC (mM)	3.05 ± 0.11	4.71 ± 0.25*	4.66 ± 0.26*
FFA (mM)	0.51 ± 0.03	1.67 ± 0.04*	1.23 ± 0.04* ^,^ #
Adiponectin (μg/ml)	11.6 ± 1.5	8.0 ± 1.6*	12.7 ± 2.1#
HDL (mM)	1.59 ± 0.15	1.47 ± 0.12	1.46 ± 0.14
LDL (mg/dl)	49 ± 11	55 ± 15	46 ± 10
Amylase (U/L)	2,220 ± 160	2,450 ± 260	2,190 ± 350
Ala aminotransferase (U/L)	320 ± 71	557 ± 103*	354 ± 89#
Asp aminotransferase (U/L)	491 ± 90	560 ± 78	457 ± 62
Creatinine (mg/dl)	0.47 ± 0.03	0.53 ± 0.04	0.54 ± 0.04
TNF‐α (pg/ml)	31.4 ± 3.9	58.2 ± 5.1*	37.1 ± 4.5#
IL‐6 (pg/ml)	10.8 ± 2.2	16.4 ± 3.5*	14.1 ± 3.1*

Note

Data are expressed as mean ± *SD*.

FFA: free fatty acid; HDL: high‐density lipoprotein; LDL: low‐density lipoprotein; TC: total cholesterol; TG: triglycerides.

* *p < *0.05 versus NC.

^#^ *p* < 0.05 versus HFD.

As mentioned above, alogliptin intervention preferred to increase heat production and reduced body weight, we further investigated its effects on body fat distribution. HFD mice had a 2.6‐fold increase in abdominal adipose deposition than NC mice, which was attenuated by alogliptin intervention (Figure 2b,c). Moreover, alogliptin intervention decreased TNF‐α and IL‐6 expression in abdominal visceral adipose tissue (Supporting Information Figure S3b).

Because liver plays a central role in regulating substance and energy metabolism, we then examined the histology of liver in various groups. High‐fat diet obviously increased the mass (Supporting Information Figure S3c) and size (Figure 2d) of liver, and alogliptin intervention prevented these alterations. Hematoxylin and eosin (H&E) and oil red O staining revealed a loss of cellular integrity and an accumulation of small‐and‐large mixed typed lipid droplets in the liver of HFD mice but not in AHF mice (Figure 2d).

Osteoporosis is a metabolic disorder of skeleton that affects millions of the elder (Liu, et al., 2016). Thus, we analyzed micro‐architectural properties of tibias by microcomputed tomography (micro‐CT). As expected, alogliptin intervention remarkably elevated trabecular relative volume (Tb.BV/TV), trabecular number (Tb.N), cortical bone mean density (BMD), and cortical thickness (Ct.Th). All of these parameters declined sharply in HFD group. Conversely, trabecular separation (Tb.Sp) and endosteal perimeter (Es.Pm) were lower in AHF group, these parameters increased in HFD group (Supporting Information Figure S4a,b).

Based on improved PWV and vasodilation function in AHF mice, we subsequently explored the morphology modifications of aortic elastic lamina. H&E (Supporting Information Figure S3d) and elastica van Gieson (VG; Supporting Information Figure S4c) staining showed the elastic lamina of thoracic aortas in HFD mice was straighter and more loose compared with NC samples, while alogliptin intervention, in some degree, preserved the wavy elastic fibers and retarded the loss of elastic density.

### 2.4 Preserved mitochondrial function

Previous study (Aroor et al., 2015) showed DPP‐4 inhibitor improved mitochondrial function, and we found alogliptin intervention reduced body weight, induced energy expenditure, and decreased abdominal adipose deposition. Thus, we wondered whether this drug increased mitochondrial biogenesis and (or) elevated mitochondrial respiration function.

**Figure 3:**
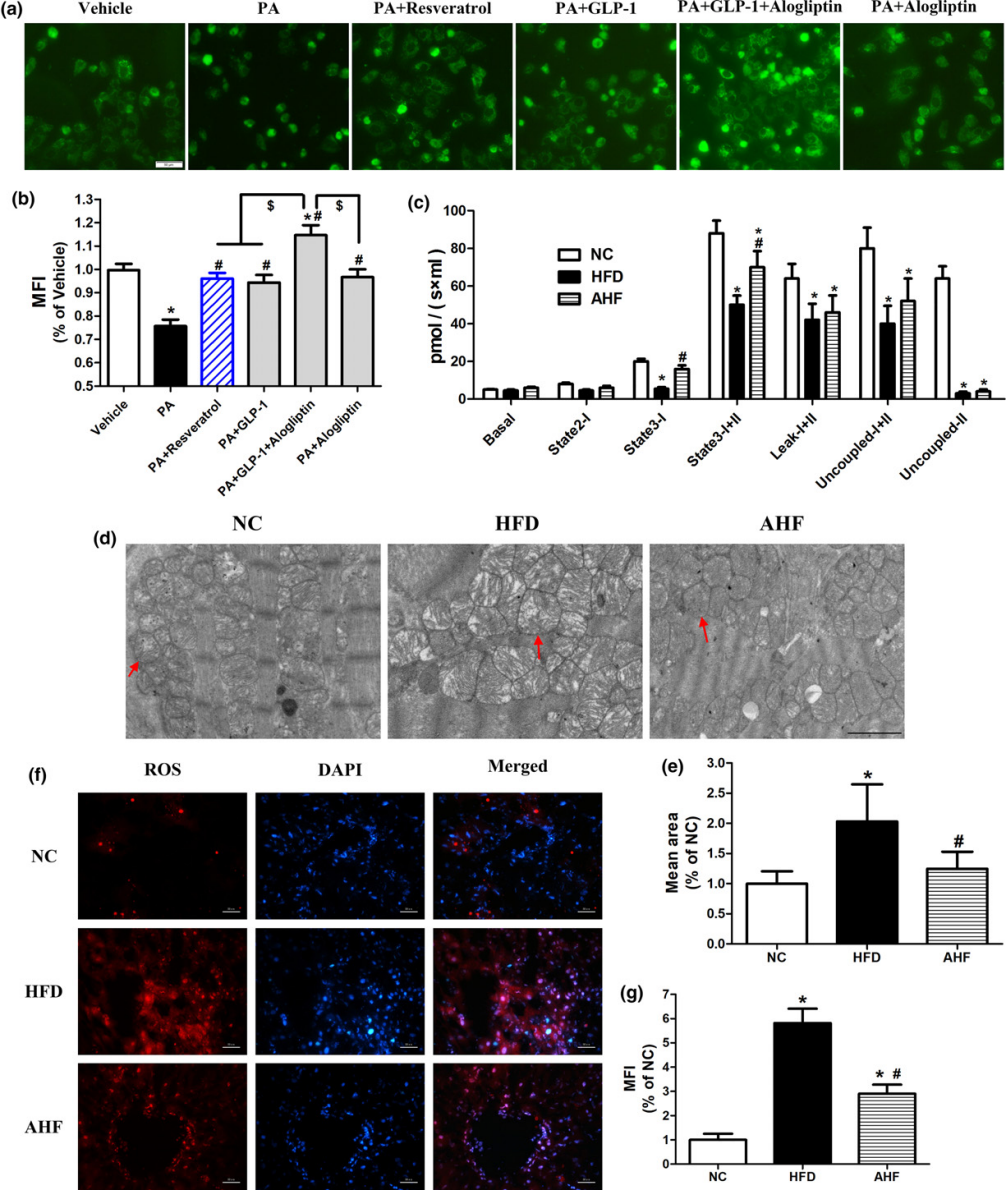
Alogliptin intervention preserves mitochondrial function. (a) Primary hepatocytes were pretreated with PA (0.4 mM) for 24 hr, following added Alog (50 µM) or resveratrol (50 µM) for 30 min, finally added GLP‐1 (100 nM), if indicated, and incubated for another 24 hr without changing culture medium. Mitochondrial number was stained with Mitotracker Green FM. Scale bar, 50 µm. (b) Quantitative analysis of (a). (c) Oxygen consumptions at different mitochondrial stages of liver. (d) Representative transmission electron micrographs of heart. The red arrows indicate mitochondria. Scale bar, 2 µm. (e) Quantitative analysis of mitochondria mean area in (d). (f) Representative images of liver sections stained for ROS (red), the nuclei were stained with DAPI (blue). Scale bar, 50 µm. (g) Quantitative analysis of (f). MFI, mean fluorescence intensity; Basal, basal respiration; State 2‐I, state 2 respiration of Complex I; State 3‐I, state 3 respiration of Complex I; State 3‐I + II, state 3 respiration of both complex I and complex II; Leak‐I + II, leak respiration; Uncoupled‐I + II, uncoupling respiration; Uncoupled‐II, uncoupling respiration of complex II. NC, normal chow. HFD, high‐fat diet. AHF, 0.03% (wt/wt) alogliptin plus HFD. Data are expressed as mean ± *SD*. *p < 0.05 compared with NC, ^#^ *p < *0.05 compared with HFD, ^$^ *p < *0.05. Each experiment repeated five times

We firstly evaluated the potential effects of alogliptin administration on mitochondrial number. To mimic internal environments, we added exogenous GLP‐1 in cell experiments. Culturing primary hepatocytes in the presence of palmate acid (PA) significantly decreased mitochondrial number, while GLP‐1, alogliptin, and resveratrol (a positive control reagent induces mitochondrial biogenesis) reversed this decrease, respectively. Furthermore, GLP‐1 and alogliptin co‐treatment played additive effects on increasing mitochondria number (Figure 3a,b). In parallel, alogliptin intervention prevented mitochondrial swelling presented in the heart of HFD mice (Figure 3d,e).

Next, we assessed mitochondrial respiration function in the liver using high‐resolution respirometry. The parameters of mitochondrial oxygen consumption including basal respiration (Basal), state 2 respiration of Complex I (State 2‐I), state 3 respiration of Complex I (State 3‐I), state 3 respiration of both complex I and complex II (State 3‐I + II), leak respiration (Leak‐I + II), uncoupling respiration (Uncoupled‐I + II), and uncoupling respiration of complex II (Uncoupled‐II) were measured. Mitochondrial oxygen consumption in all statuses elevated in the liver of AHF mice (Figure 3c and Supporting Information Figure S5), indicating improved mitochondrial respiratory function by alogliptin intervention. Furthermore, we found ROS production was dramatically higher in the liver of HFD group compared with NC group and alogliptin intervention significantly decreased it (Figure 3f,g), suggesting that alogliptin intervention reduced oxidative stress.

### 2.5 Changes of transcriptome

**Figure 4:**
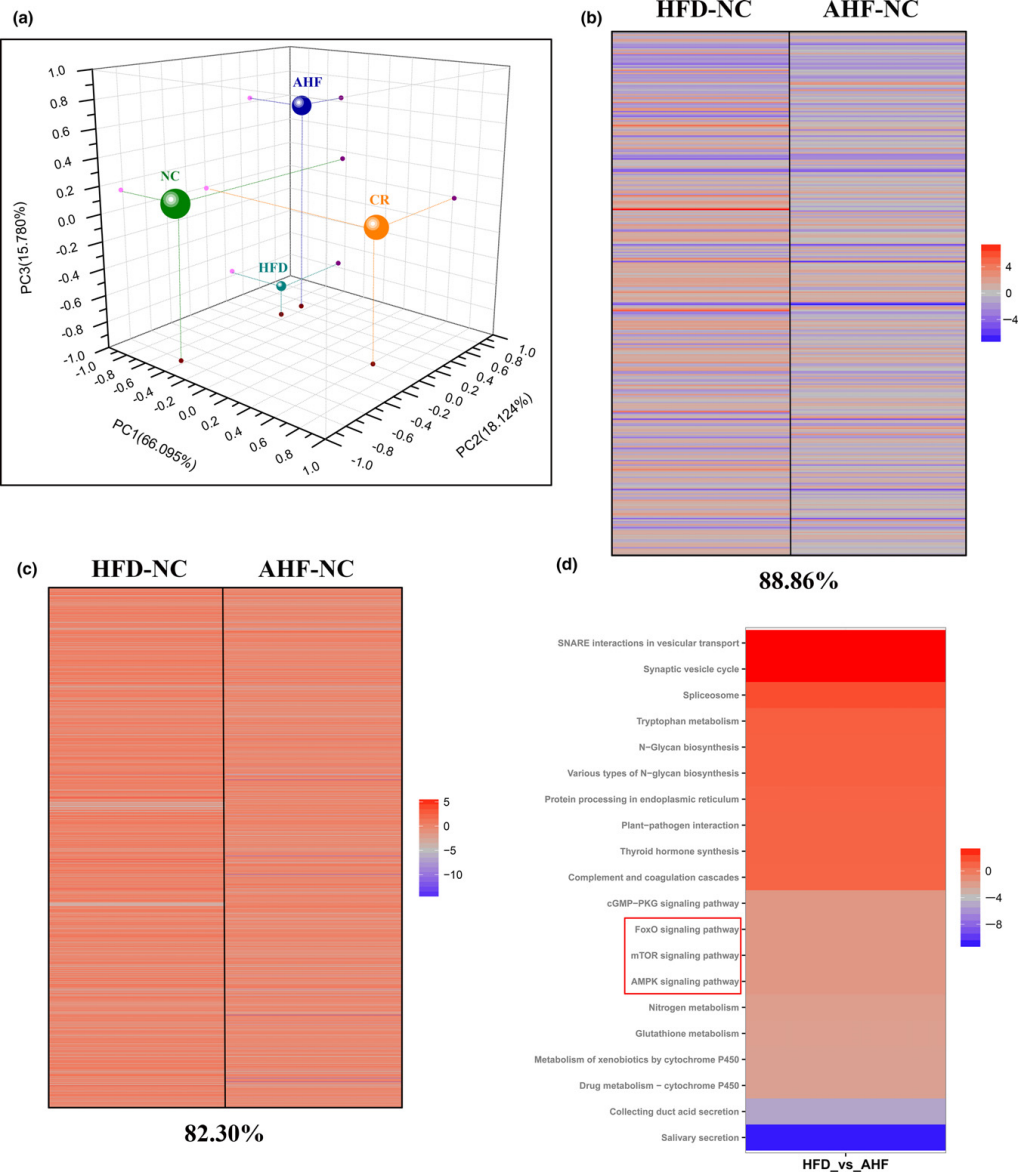
Changes of transcriptome in the liver. (a) Principal component analysis (PCA) was performed based on differentially expressed genes from the liver. Each data point corresponds to the PCA analysis of each group. (b) Gene expression profile comparing genes significantly upregulated (red) and downregulated (blue) by either AHF or HFD compared with NC mice (fold‐change), and the complete list of significantly altered genes can be found in Supporting Information Table S1. The percentage of significant gene expression changes shifted in the same direction in AHF and HFD compared with NC mice is presented at the bottom of the figure. (c) Comparison of gene sets significantly altered by AHF and HFD treatment compared with NC expression (fpkm); upregulated (red) and downregulated (blue) gene sets. The complete list of significantly altered gene sets can be found in Supporting Information Table S2. The percentage of significant gene sets changes shifted in the same direction in AHF and HFD compared with NC is presented at the bottom of the figure. (d) Top 20 of the most up‐ and down‐regulated signaling pathways selected by Kyoto Encyclopedia of Genes and Genomes (KEGG) pathway analysis. NC, normal chow. HFD, high‐fat diet. AHF, 0.03% (wt/wt) alogliptin plus HFD. 3 mice per group

To get a deeper understanding of molecular mechanisms elicited by alogliptin intervention, high‐throughput RNA sequencing (RNA‐seq) of expression level of all genes in the liver was performed. Because caloric restriction (CR) is the most reproducible way to delay age‐related diseases and extend lifespan in diverse species (Baur et al., 2006), we firstly tested whether alogliptin intervention shifted the physiology toward the mice on CR. Principal component analysis (PCA) showed the global gene expression profile of alogliptin intervention shifted distant from alterations induced by CR (Figure 4a), indicating alogliptin intervention could not mimic CR transcriptome.

We further highlighted the transcript profiles and found 11.14% of genes expression was modified by alogliptin intervention in the different direction from HFD (Figure 4b). In parallel, we analyzed gene sets modified in response to alogliptin intervention. Results showed alogliptin intervention made a 17.70% change of upregulated and downregulated gene sets disparate from HFD (Figure 4c).

**Figure 5:**
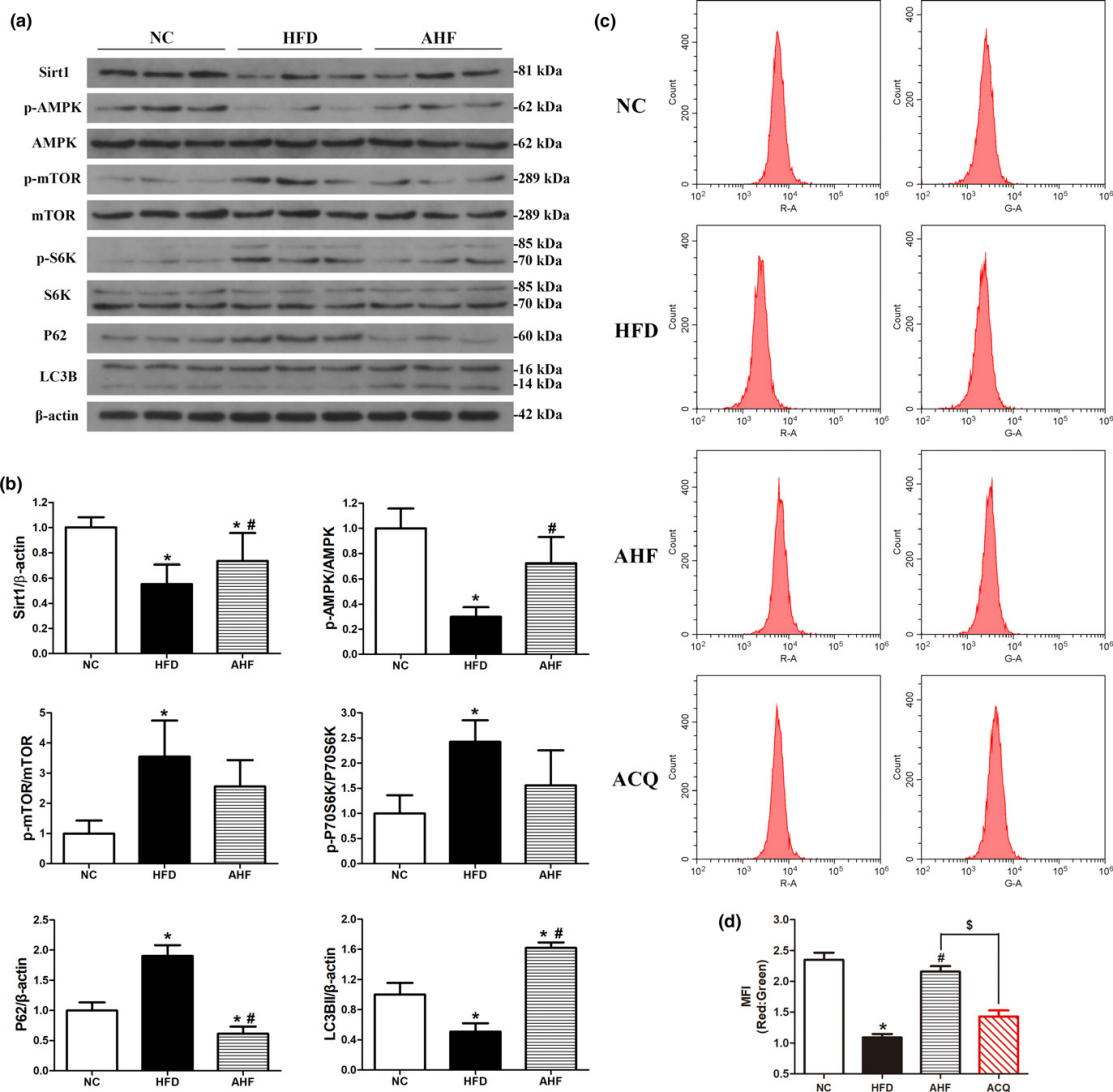
Alogliptin intervention activates autophagy in vivo. (a) Western blots of total liver lysates for Sirt1, phosphorylated (p)‐AMPK, AMPK, p‐mTOR, mTOR, p‐S6K, S6K, p62, LC3B (LC3BII, 14 kDa). β‐Actin was used as a loading control. (b) Quantitative analysis of (a). (c) Mice were injected with adenovirus‐encoding mRFP‐GFP‐LC3 seven days before the end of the study, then the liver cells were isolated and assessed by flow cytometry. (d) Quantitative analysis of (c). MFI, mean fluorescence intensity. NC, normal chow. HFD, high‐fat diet. AHF, 0.03% (wt/wt) alogliptin plus HFD. AHQ, AHF plus CQ (chloroquine; 60 mg/kg, i.p. injection 6 hr before sacrificing the mice). Data are expressed as mean ± *SD*. *p < 0.05 compared with NC, ^#^ *p < *0.05 compared with HFD, ^$^ *p < *0.05

Finally, we performed Kyoto Encyclopedia of Genes and Genomes (KEGG) pathway analysis to search for the signaling pathways of interest involved in the effects of alogliptin intervention on promoting lifespan and healthspan. Results indicated alogliptin intervention regulated the expression of genes associated with FOXO (fork‐head box O), AMPK (adenosine monophosphate‐activated protein kinase), and mTOR (mammalian target of rapamycin) signaling pathways (Figure 4d). It is widely admitted insulin/insulin‐like growth factor 1 (IGF‐1)‐FOXO signaling pathway was associated with longevity. Subsequently, we tested this pathway and results showed alogliptin treatment had no influence on the expressions of p‐FOXO1 and p‐FOXO3a (Supporting Information Figure S6). AMPK is a master energy sensor and mTOR is an evolutionarily conserved regulator of cellular growth and metabolism. In parallel, we confirmed these pathways and found increased AMPK phosphorylation and decreased mTOR phosphorylation in AHF group compared with HFD group (Figure 5a), suggesting alogliptin intervention activated AMPK and inhibited mTOR.

### 2.6 Upregulated autophagy in vivo

Since AMPK‐mTOR signaling pathway is related to longevity and modulates autophagy, autophagy activation also contributes to delaying aging process (Vilchez, Saez, & Dillin, 2014). Thus, we next explored whether alogliptin intervention induced autophagy. Analysis of liver protein levels of microtubule‐associated light chain 3B‐II (LC3B‐II) revealed alogliptin intervention significantly increased relative LC3B‐II levels, which were decreased in HFD mice, indicating an accumulation of autophagosome. To distinguish activated autophagy from blocked fusion of autophagosome and lysosome leading to increased LC3B‐II, we also measured sequestosome‐1 (p62) protein levels. Results showed p62 level was decreased in AHF group, while accumulated in HFD group, indicating autophagy level was higher in AHF group (Figure 5a,b). However, p62 might also degrade via ubiquitin‐proteasome pathway leading to decreased p62. Therefore, we used chloroquine (CQ), a specific autophagy inhibitor blocking lysosomal acidification, to further clarify the autophagy level in AHF mice. We also measured autophagic flux by delivering mRFP‐GFP‐LC3 to the lysosome when only GFP proteins quenched in low pH [autophagosomes were labeled with yellow signal (mRFP‐GFP) and autolysosomes with red signal (mRFP)]. In accordance with Western blotting results, mean fluorescence intensity (MFI) of red/green remarkably reduced in HFD mice compared to NC mice, indicating decreased autophagy flux, and autophagy flux significantly increased by alogliptin intervention, while MFI of red/green declined sharply after CQ administration (Figure 5c,d). Taken together, these results suggested alogliptin intervention truly induced autophagy on high‐fat diet.

Autophagy is regulated by mTOR‐dependent and mTOR‐independent signaling pathways (Vilchez et al., 2014). Given that activated AMPK directly inhibits mTOR and thereafter induces autophagy (Rubinsztein, Marino, & Kroemer, 2011). We further explored these signaling pathways (Figure 5a,b) and found AMPK phosphorylation was increased in the liver of AHF mice. Consequently, mTOR and p70‐ribosomal S6 protein kinase (p70‐S6K) phosphorylation levels decreased, indicating mTOR and p70‐S6K were inhibited in AHF mice and subsequently induced autophagy. Regarding mTOR‐independent pathways, sirtuin 1 (Sirt1) could also mediate autophagy via deacetylation of several autophagy‐related (Atg) proteins and transcription factors (Lee et al., 2008; Vilchez et al., 2014). Here, we showed alogliptin intervention significantly elevated Sirt1 protein level, which decreased on long‐term high‐fat diet.

Since AMPK could also directly phosphorylate ULK1(unc‐51‐like kinase 1) to initiate autophagy, whereas mTOR negatively regulates ULK1 by phosphorylation of Ser757 to inhibit autophagy (Gui et al., 2017; Li et al., 2018). Thus, we explored whether AMPK directly activated autophagy independent of the inhibition of mTOR. Results indicated AMPK‐ULK1 pathway did not involve in autophagy activation by alogliptin intervention (Supporting Information Figure S7a,b).

### 2.7 Activated autophagy in vitro

**Figure 6:**
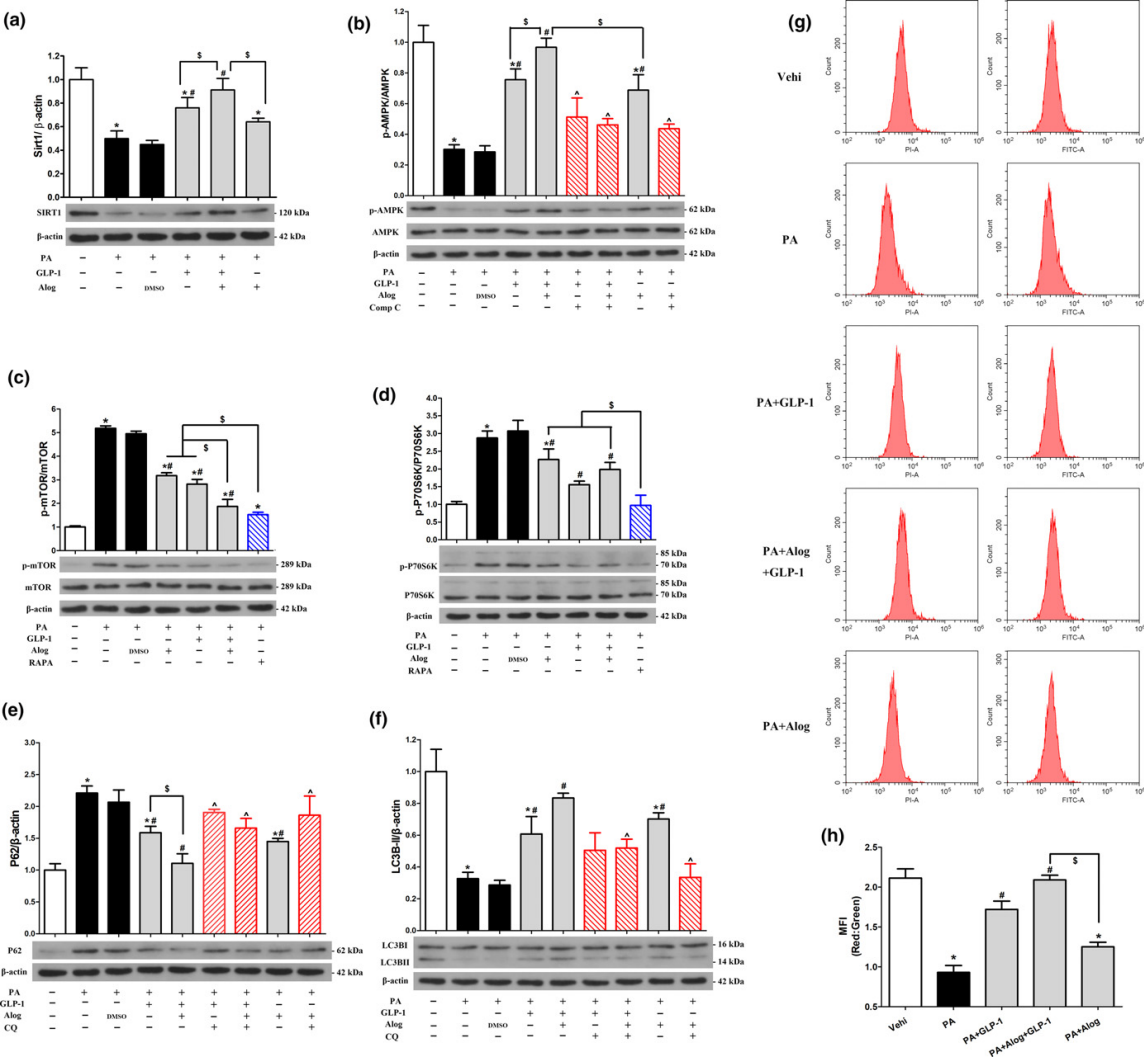
Alogliptin administration stimulates autophagy in vitro. (a–f) Primary hepatocytes were pretreated with PA (0.4 mM) for 24 hr, and then added inhibitors [Compound C (20 µM), RAPA (1 µM) or CQ (10 µM)] for 30 min, following added Alog (50 µM) or DMSO (solvent of Alog) for 30 min, finally added GLP‐1 (100 nM) where indicated. After incubation without changing culture medium for another 24 hr, cells were harvested for Western blotting. Representative immunoblots and densitometric quantification for the expression of proteins Sirt1 (a), phosphorylated (p)‐AMPK (b), p‐mTOR (c), p‐S6K (d), p62 (e), and LC3B (LC3BII, 14 kDa) (f). (g‐h) Primary hepatocytes were pretreated with PA (0.4 mM) for 24 hr, and then added Alog (50 µM) for 30 min, following added GLP‐1 (100 nM), if indicated. Then, the cells were incubated without changing culture medium for another 24 hr. Adenoviruses encoding mRFP‐GFP‐LC3 were transfected 48 hr prior to harvest. (g) Representative graphs of flow cytometry. (h) Quantification of (g), *p < 0.05 compared with the Vehi (vehicle) group, ^#^ *p* < 0.05 compared with PA group, ^^^ *p* < 0.05 compared with corresponding groups without inhibitors, ^$^ *p* < 0.05. PA, palmitic acid. RAPA, rapamycin. CQ, chloroquine. Data are expressed as mean ± *SD*. Each cell experiment repeated five times

We next explored the effects of alogliptin administration on autophagy in primary hepatocytes. Results implied p62 proteins levels significantly decreased and LC3B‐II correspondingly increased in the presence of GLP‐1, alogliptin, GLP‐1 and alogliptin co‐treatment, respectively. In addition, GLP‐1 and alogliptin co‐treatment had additive effects on decreasing p62 and elevating LC3B‐II. Their effects were blunt by addition of CQ (Figure 6e,f). In accordance with the results of Western blotting, MFI of red/green increased in GLP‐1 and alogliptin administration compared with PA group, respectively. Similarly, the effect of GLP‐1 and alogliptin co‐treatment was more noticeable (Figure 6g,h). We also obtained similar results in LC3B turnover assay using bafilomycin A1 (Supporting Information Figure S8i,j). Additionally, to determine whether alogliptin administration stimulated autophagy via inhibiting DPP‐4, we used DPP‐4 small interfering RNA (siRNA) to suppress DPP‐4 and explored its effects on autophagy flux. Results showed DPP‐4 inhibition alone had no influence on autophagy flux (Supporting Information Figure S9).

To confirm the signaling regulating autophagy in vitro, we also explored mTOR‐dependent and mTOR‐independent pathways. Results revealed mTOR (Figure 6c) and its downstream molecular p70‐S6K (Figure 6d) were inhibited by adding GLP‐1, alogliptin, and combining use of GLP‐1 and alogliptin, respectively. Interestingly, the inhibition effects of GLP‐1 and alogliptin co‐treatment were even close to rapamycin. Simultaneously, we detected increased AMPK phosphorylation by addition of GLP‐1, alogliptin, and GLP‐1 and alogliptin co‐treatment, respectively, which was significantly blocked by Compound C (Figure 6b). Regarding mTOR‐independent pathway, we also found GLP‐1, alogliptin, and GLP‐1 and alogliptin co‐treatment increased Sirt1 protein levels, respectively (Figure 6a). Moreover, GLP‐1 and alogliptin co‐treatment also had additive effects on elevating expression of Sirt1 and p‐AMPK, reducing p‐mTOR and p‐p70S6K protein levels. In line with primary hepatocytes model, alogliptin administration also activated autophagy in L‐02 cells (Supporting Information Figure S8a–h). In accordance with in vivo study, AMPK‐mTOR‐ULK1 pathway involved in autophagy activation in vitro experiments (Supporting Information Figure S7c).

### 2.8 Autophagy is required for improving health and extending longevity

To determine whether the improved health and extended lifespan of alogliptin intervention on high‐fat diet depend on autophagy, we inhibited autophagy by delivering Atg7 siRNA to AHF mice (experiment group, Exp). Firstly, we confirmed knockdown Atg7 in the liver of Exp mice compared with Con (control group) mice (Supporting Information Figure S10a). Atg7 was necessary for autophagosome formation, consequently, we observed significantly increased p62 and decreased LC3B‐II proteins in the liver of Exp mice (Supporting Information Figure S10b). Subsequently, we found the attenuated lipid accumulation by alogliptin intervention was disappeared in Exp mice (Supporting Information Figure S10d). In parallel, abdominal adipose deposition reduced in Con mice but not in Exp mice (Supporting Information Figure S10c). Secondly, the Exp mice displayed decreased glucose tolerance (Supporting Information Figure S11a) and insulin sensitivity (Supporting Information Figure S11b) as well as reduced β‐cell proportion (Supporting Information Figure S11c). Thirdly, improved endothelium‐independent vasodilation function of aortas was observed in Con mice but disappeared in Exp mice (Supporting Information Figure S12a). In addition, the elastic lamina of thoracic aortas in Exp mice also seemed to be straighter (Supporting Information Figure S12b). Finally, we detected decreased complex activity of electron transport chain (ETC) in mitochondrial respiration in the liver of Exp mice (Supporting Information Figure S13). Notably, the longevity‐extending effects of alogliptin intervention markedly diminished when Atg7 knockdown (*χ* ^2^ = 5.308, *p* = 0.021; Supporting Information Figure S14). Collectively, these data indicated alogliptin intervention improved age‐related diseases and promoted longevity, at least partly, through activating autophagy.

## 3 DISCUSSION

Our present data for the first time suggested that alogliptin intervention extends longevity and improves health of mice with excess caloric intake. These beneficial effects are mainly related to increased insulin sensitivity, attenuated function decline, decreased organ pathology, inhibited inflammation, preserved mitochondrial function, and reduced oxidative stress. Autophagy activation may be involved in these beneficial effects. Thus, alogliptin intervention is considered as a potential strategy for extending lifespan and healthspan in obesity and overweight.

A growing body of evidence (Aroor et al., 2015; Matsubara et al., 2012; Meng et al., 2016; Moritoh et al., 2008; Zhuge et al., 2016) suggested short‐term treatment with DPP‐4 inhibitors and GLP‐1 analogues attenuated a broad spectrum of age‐related diseases. In present study, we proved long‐term alogliptin intervention produced similar protective effects on age‐associated diseases. Especially, as expected, long‐term alogliptin treatment extended longevity and improved healthspan on high‐fat diet. Additionally, our study showed sitagliptin, another DPP‐4 inhibitor which is structurally distant from alogliptin (Wang et al., 2016), took a similar longevity‐promoting effect. Based on these data, we speculate the life‐extending effect is a class effect of DPP‐4 inhibitors.

Our RNA‐seq analysis results indicated autophagy may be one of the key mechanisms linked to these beneficial effects of alogliptin intervention. In our study, over‐time high‐fat diet declined basal level of autophagy, leading to an accumulation of unnecessary and damaged substrates, particularly impaired mitochondria. The accumulation of autophagy cargos impaired cell function and tissue homeostasis, ultimately resulted in multiple age‐associated diseases and reduced lifespan. Conversely, autophagy activation by alogliptin improved health and extended longevity. Firstly, we demonstrated alogliptin administration activated autophagy both in vivo and in vitro. In in vivo experiment, CQ decreased the higher level of autophagy flux activated by alogliptin, providing disproof of activated autophagy in AHF mice. In in vitro experiment, DPP‐4 inhibition using siRNA had no influence on autophagy flux, suggesting alogliptin also directly stimulated autophagy independent of inhibiting DPP‐4. Together, we found alogliptin intervention activated autophagy, which might take beneficial effects on lifespan extension. These results are congruent with several previous studies (Bjedov et al., 2010; Harrison et al., 2009; Kenyon, 2010; Pyo et al., 2013; Vilchez et al., 2014) showing either genetical or pharmacological activation of autophagy extended lifespan in devious species. Secondly, it has been proposed autophagy is necessary for maintaining mitochondrial functions (Choi et al., 2017). Moreover, impaired mitochondrial functions increased ROS production and excess ROS led to oxidative stress (Mitchell et al., 2016). In present study, alogliptin increased mitochondrial biogenesis, elevated mitochondrial respiration function and prevented mitochondrial swelling, consequently reduced ROS production. Conversely, the elevated respiration in alogliptin intervention decreased sharply when inhibiting autophagy using Atg7 siRNA. Additionally, in liver, mitochondrial biogenesis is positively regulated by Sirt1 (Baur et al., 2006). We detected increased Sirt1 protein levels after alogliptin administration both in vivo and in vitro. Collectively, alogliptin preserved mitochondrial functions, which may also contribute to extending lifespan. Finally, when autophagy was inhibited, the attenuated accumulation of lipid drops disappeared; the improved endothelium‐independent vasodilation function significantly decreased; the ameliorated state of insulin resistance and preserved β‐cell proportion were blunted. More importantly, the extended lifespan by alogliptin intervention reduced in old mice. These data suggested autophagy, at least partly, mediated the improvement of age‐related diseases and lifespan extension.

Next, we explored the signaling pathways that regulating autophagy. It is well known autophagy is mainly regulated by mTOR‐dependent pathway, and AMPK is an important upstream regulator of mTOR. Furthermore, AMPK crosses talk with Sirt1, and Sirt1 also mediates autophagy via mTOR‐independent pathways. Here, we demonstrated alogliptin administration increased Sirt1 protein level and activated AMPK both in vivo and in vitro. Consequently, mTOR was inhibited after alogliptin administration and thereafter activated autophagy. In in vitro experiments, we found not only GLP‐1 but alogliptin increased Sirt1 protein level, activated AMPK, inhibited mTOR, and consequently stimulated autophagy. Moreover, GLP‐1 and alogliptin co‐treatment took additive effects. These effects may be partly due to DPP‐4 inhibition by alogliptin and consequently elevated GLP‐1 level. Importantly, as mentioned above, in our in vitro experiments, alogliptin alone directly activated Sirt1 and AMPK, and subsequently inhibited mTOR independent of elevating GLP‐1. Cumulatively, these results suggested autophagy activation after alogliptin administration, at least partially, depend on the Sirt1‐AMPK‐mTOR cascade.

In addition, our RNA‐seq analysis results also showed FOXO signaling pathway was significantly upregulated after alogliptin intervention. However, in our animal study, long‐term alogliptin treatment did not change the expressions of p‐FOXO1 and p‐FOXO3a. Furthermore, plasma levels of IGF‐1 in AHF mice did not significantly differ from HFD mice. These data suggested FOXO signaling pathway actually did not involve in the benefit effects of alogliptin intervention on health and longevity.

Our study has some limitations. First, we did not explore the effects of DPP‐4 activity itself on lifespan in vivo. Although we provided in vitro evidences showing DPP‐4 inhibition alone did not stimulate autophagy, but still could not rule out DPP‐4 inhibition itself might take beneficial effects on improving health and extending longevity in vivo. If DPP‐4 was knocked out or knocked down in vivo, it would subsequently elevate GLP‐1 and glucose‐dependent insulinotropic polypeptide (GIP) levels. Then, we could not distinguish the beneficial effects of incretins from DPP‐4 inhibition itself. In addition, DPP‐4 inhibition in vivo also worked on other respects, such as immunoregulation by affecting leukocyte migration (Shah et al., 2011), which might also contribute to improving health and survival. Second, our animal model is obesity and overweight, so it is still not known whether our results are applicable to normal weight animals. Further studies with additional animal models are needed to generalize the effects of alogliptin intervention to normal individuals.

In conclusion, our present data demonstrate that alogliptin intervention is efficacious on improving survival and health of mice on a high‐fat diet and we consider these beneficial effects as a comprehensive result. Here, autophagy is proposed as an underlying reason of these beneficial effects. In addition, multiple beneficial effects, including anti‐insulin resistance, anti‐obesity, cardiovascular protection, and anti‐aging, were observed by alogliptin intervention in HFD‐fed mice. Because, our animal model in this study is metabolic syndrome, so we consider anti‐insulin resistance may be more primary.

## 4 EXPERIMENTAL PROCEDURES

More detailed methods are described in the Supporting Information Appendix S1.

### 4.1 Animals models and diets

Animal procedures conformed to the National Institutes of Health Guidelines for the Use of Laboratory Animals and were approved by the Animal Ethics Committee of Wuhan General Hospital. For dose–response experiment of alogliptin treatment, male ApoE^−/−^ mice at 12 months of age were randomly assigned to the following diets for the remainder of their lives: HFD (high‐fat diet, normal chow modified to provide 60% of calories from fat, *n* = 12 mice), LAHF [low dose of alogliptin (0.01% wt/wt) plus HFD, *n* = 13 mice] and HAHF [high dose of alogliptin (0.03% wt/wt) plus HFD, *n* = 13 mice].

Male C57BL/6J mice at 12 months of age were maintained on normal chow (NC), HFD or 0.03% (wt/wt) alogliptin plus HFD (AHF, *n* = 55 in each group). Body weight and food intake were measured on weekly basis during the period of the study. To determine autophagy flux, we injected mice [three mice in NC, HFD, AHF, and AHQ (AHF plus CQ) groups] with adenovirus‐encoding mRFP‐GFP‐LC3 (0.1 ml; 10^11^ PFU/ml, Hanbio, China) through tail vein 7 days before the end of the study. CQ (60 mg/kg) was intraperitoneally (i.p.) administered 6 hr before sacrificing AHQ mice. At the terminal point of the study, 26‐month‐old mice were fasted overnight and then anesthetized by i.p. injection of pentobarbital sodium (60 mg/kg) and euthanized for serum and tissues samples. For the longevity study of sitagliptin, male C57BL/6J mice at 11 months of age were maintained on normal chow until they reached one year of age. Then, the chow was switched to 0.4% (wt/wt) sitagliptin plus HFD (SHF, *n* = 50 mice) and the dose was estimated from previous research (Takeda et al., 2012).

To access the effects of autophagy inhibition on longevity and health, another 12‐month‐old male C57BL/6J mice were randomly assigned to control group (Con, *n* = 55 mice) and experimental group (Exp, *n* = 55 mice). The mice were fed with AHF for their reminder lives. During the period, the mice in Exp group were injected with adenovirus‐encoding Atg7 siRNA (0.15 ml; 10^10^ PFU/ml, Hanbio, China) through the tail vein once every three weeks, and the Con mice were correspondingly injected with adenovirus‐encoding scrambled siRNA.

All mice were maintained in a specific‐pathogen‐free environment (20–22°C) with a 12 hr light/dark cycle and unrestricted access to water and food. Survival curves were plotted using the Kaplan–Meier method, which included all available animals at each time point. The death of mice was recorded on a weekly basis.

### 4.2 Histology

Tissues were fixed in 4% formaldehyde, then sectioned and stained with H&E or van Gieson (VG). The immunofluorescence staining of pancreatic tissues was performed as previously reported (Li et al., 2017). Livers were frozen at −80°C and stained with oil red O or ROS fluorescent probe‐dihydroethidium.

### 4.3 Mitochondrial mass assays

Primary hepatocytes were isolated from eight‐week‐old male C57BL/6 mice and cultured as previously described (Guo et al., 2017). Mitochondrial mass was stained as previously reported (Baur et al., 2006) using Mitotracke Green FM.

### 4.4 Mitochondrial respiratory function

Liver mitochondrial respiration was assessed using Oxygraph‐2k high‐resolution respirometry (Oroboros Instruments, Innsbruck, Austria) similar to previously reported (Aroor et al., 2015; Eisenberg et al., 2016; Li et al., 2015). Mitochondria extracted from liver tissue of 26 months old mice (*n* = 6 mice per group) were harvested. After which, substrates, uncoupler, and inhibitors were applied to analyze respiratory function.

### 4.5 Monitoring autophagic flux

Primary hepatocytes and L‐02 cells were infected 24 hr before assay with adenovirus‐encoding mRFP‐GFP‐LC3 (10^10^ PFU/ml; Hanbio, China) at 30 multiplicities of infection as previously reported (Guo et al., 2017). Then, MFI of red/green or the number of autophagosomes (yellow puncta) and autolysosomes (red puncta) per cell was calculated to measure autophagic flux.

### 4.6 RNA‐seq

Total RNA of the liver was prepared with Trizol reagent (Invitrogen) and RNA‐seq was performed by Majorbio technology Inc. (Shanghai, China). The RNA‐seq data have been submitted to Sequence Read Archive (https://www.ncbi.nlm.nih.gov/sra/) under accession number SRP127778. CR data were pre‐existed at the Gene Expression Omnibus (GEO; https://www.ncbi.nlm.nih.gov/geo/; accession number GSE71773).

### 4.7 Western blotting

Measurements of target protein levels were performed in tissues of 26 months old mice (*n* = 3 mice per group) or cell extracts. Antibodies: Sirt1, phosphorylated (p)‐AMPK, AMPK, p‐mTOR, mTOR, p‐S6K, S6K, p‐ULK1(S317), p‐ULK1(S555), p‐ULK1(S757), ULK1, p62, LC3B, p‐FOXO1, FOXO1, p‐FOXO3a, FOXO3a, Atg7 were used.

### 4.8 Other parameters

Serum biochemical markers, rotarod, echocardiography, and et al. are described in the Supporting Information Appendix S1.

### 4.9 Statistical analysis

Values are expressed as mean ± *SD* except where noted. Statistical significance was analyzed by one‐way ANOVA following with LSD post hoc tests when equal variances were assumed or Tamhane’s T2 post hoc tests when equal variances were not assumed for comparisons between two or multiple groups. Homogeneity of variance was tested using Levene’s test. The significance values are *p *< 0.05 (two‐sided). Statistical testing was performed using IBM SPSS 22.0 statistics software (IBM Corp, Armonk, NY).

## CONFLICT OF INTEREST

The authors declare no competing financial interests.

## AUTHOR CONTRIBUTIONS

B.Z., H.L., J.Z., B.G., L.W., M.L., and L.C. conducted the animal experiments. Y.L., L.X., J.D., and M.L. performed the in vitro experiments. B.Z., L.X., W.M., H.L., and B.G. analyzed the data and wrote the manuscript. G.X. is the guarantor of this work and, as such, has full access to all the data in the study and takes responsibility for the integrity of the data and the accuracy of the data analysis.
