# Supplementary figures and images for: Alogliptin improves survival and health of mice on a high‐fat diet
Source: Aging Cell. 2019 Jan 15;18(2):e12883. doi: 10.1111/acel.12883 (PMC6413659; doi:10.1111/acel.12883)

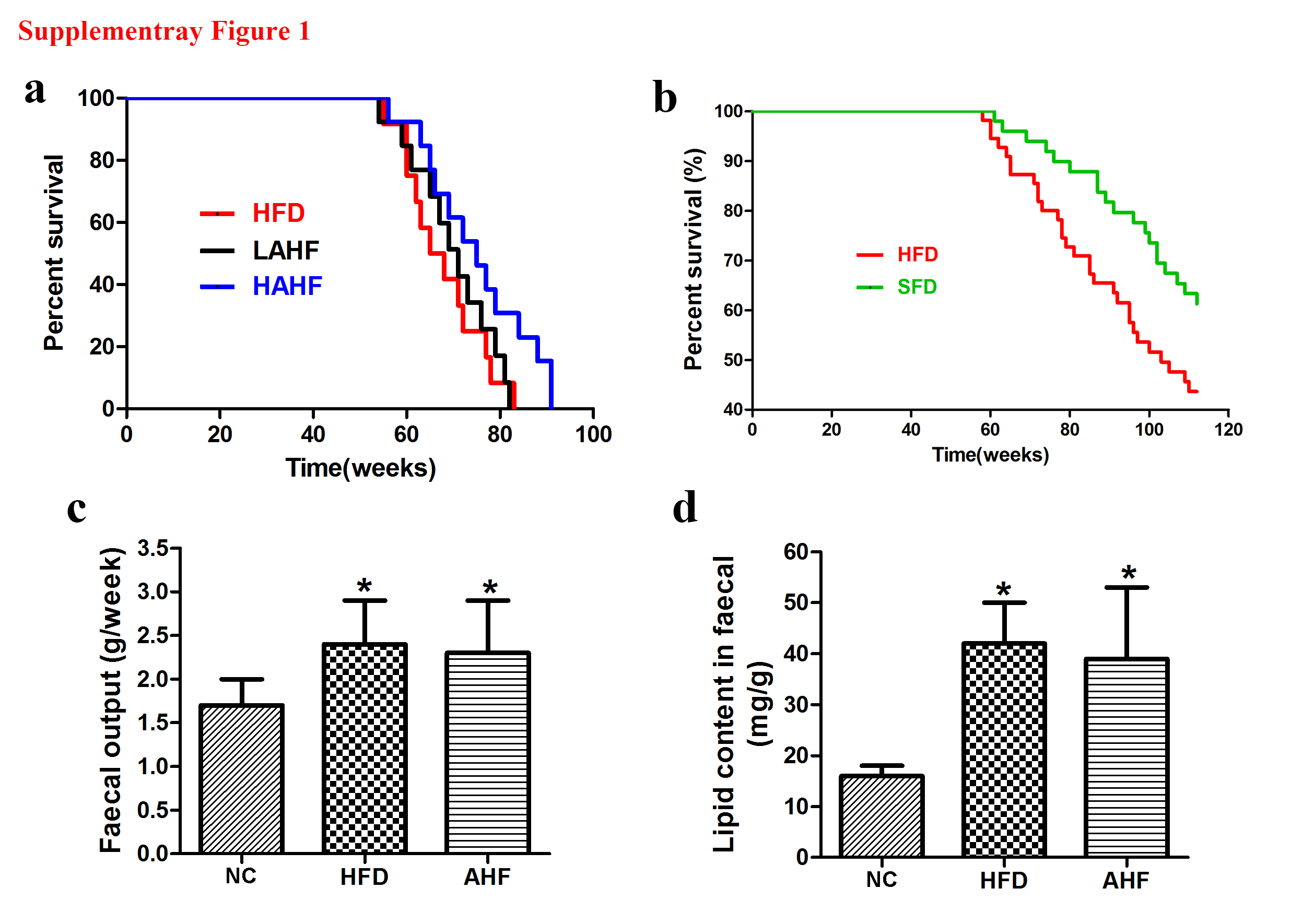

Supplement: Supplementary file 1 [file ACEL-18-e12883-s001.tif]

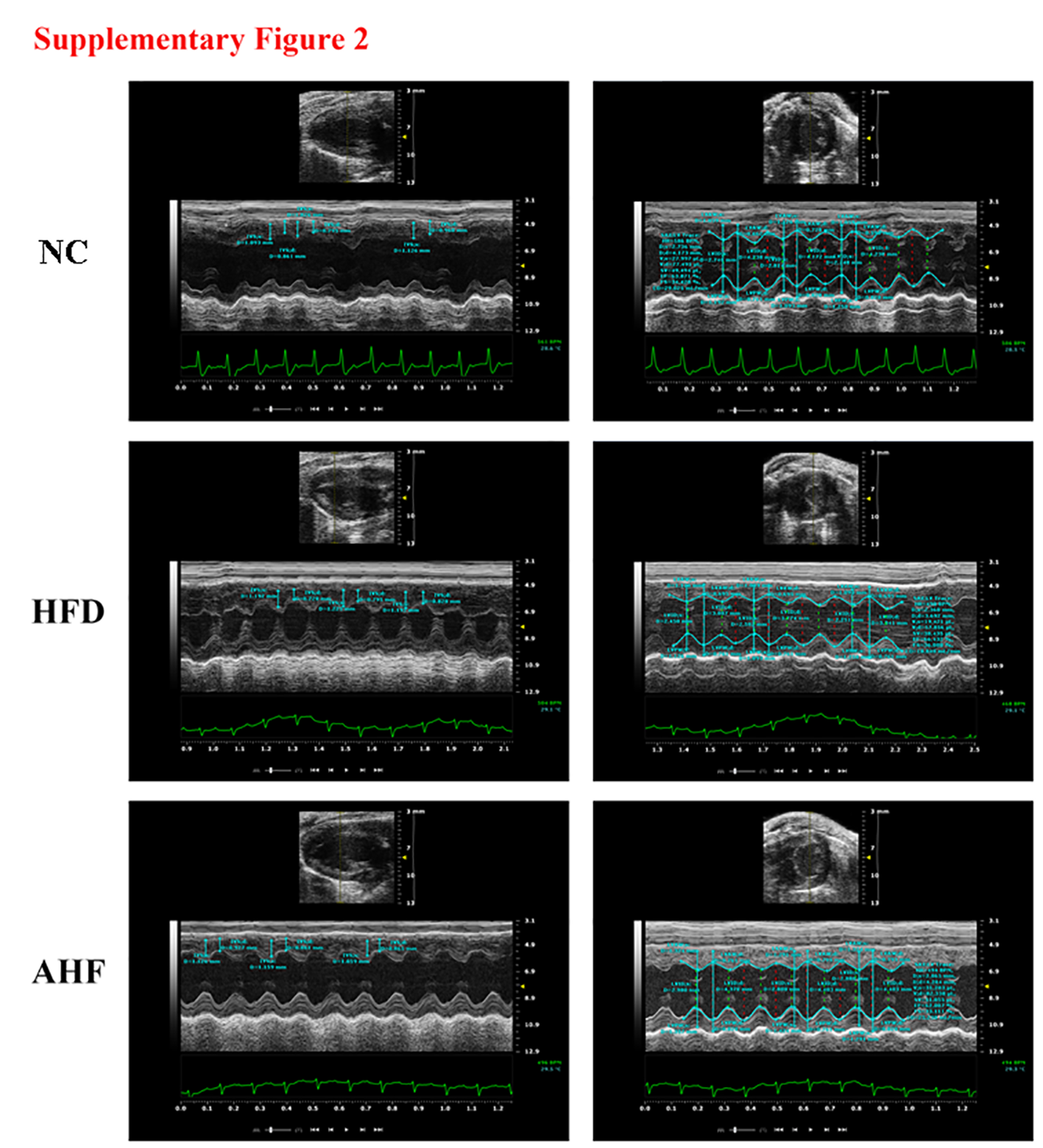

Supplement: Supplementary file 2 [file ACEL-18-e12883-s002.tif]

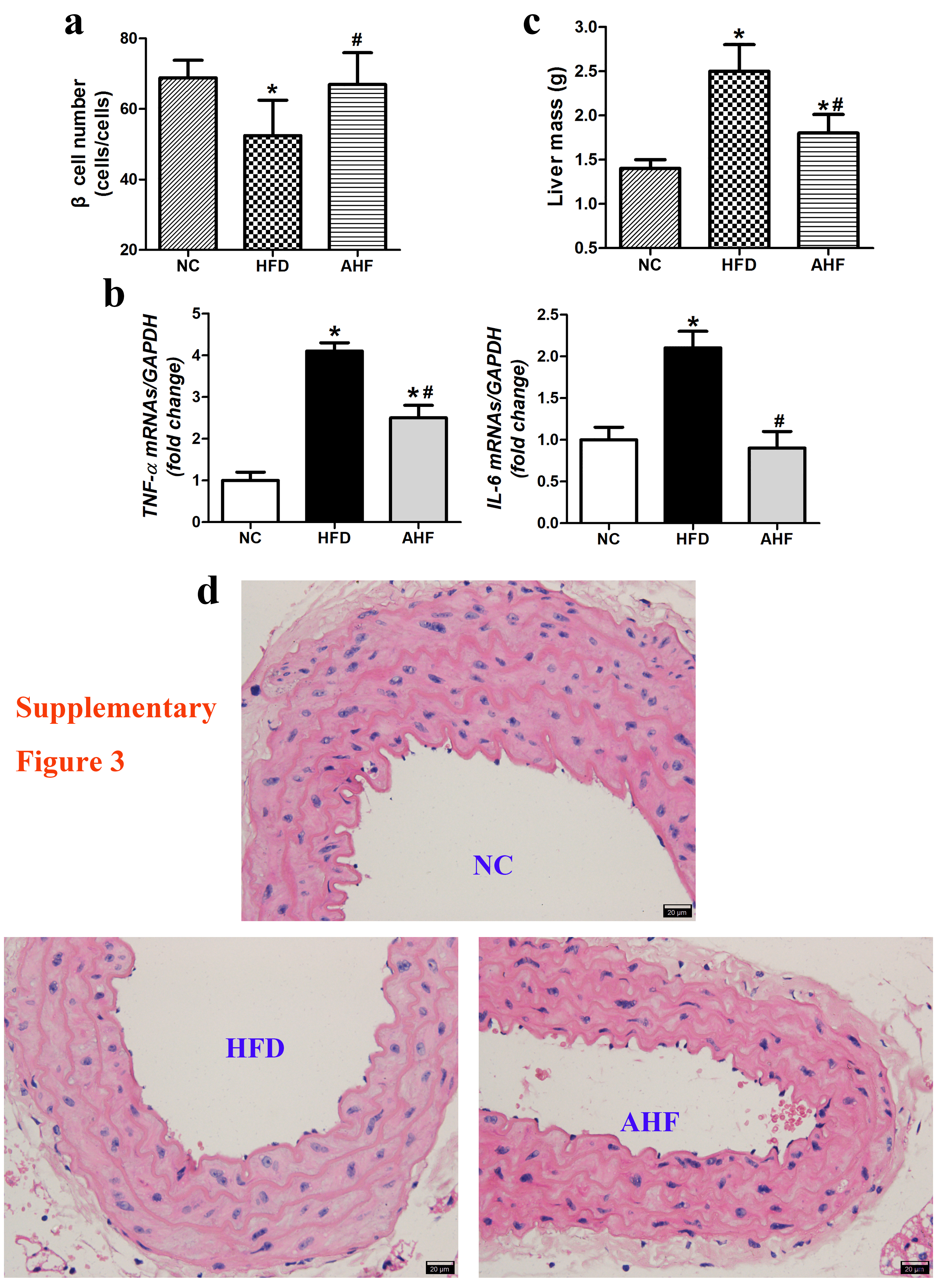

Supplement: Supplementary file 3 [file ACEL-18-e12883-s003.tif]

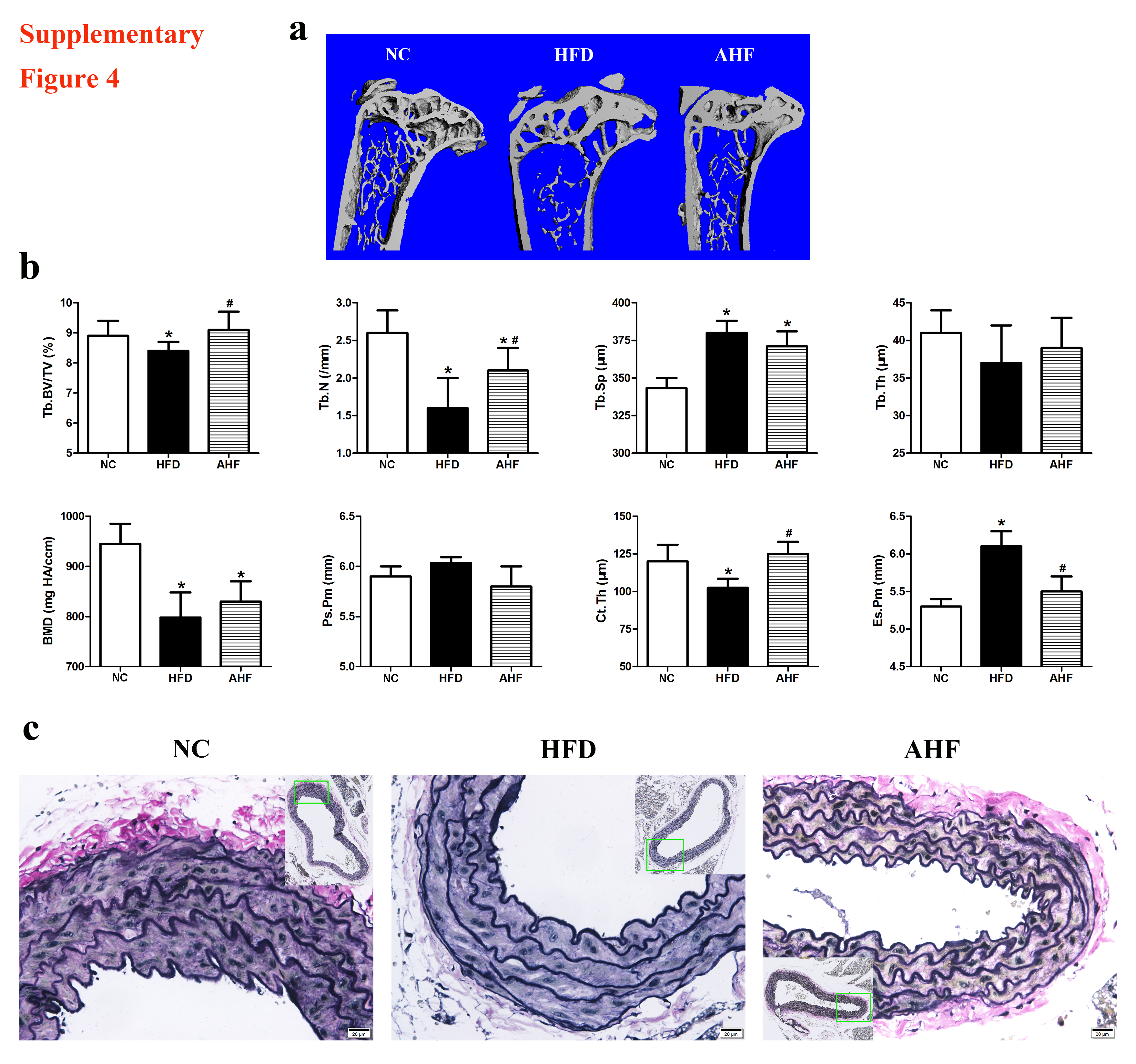

Supplement: Supplementary file 4 [file ACEL-18-e12883-s004.tif]

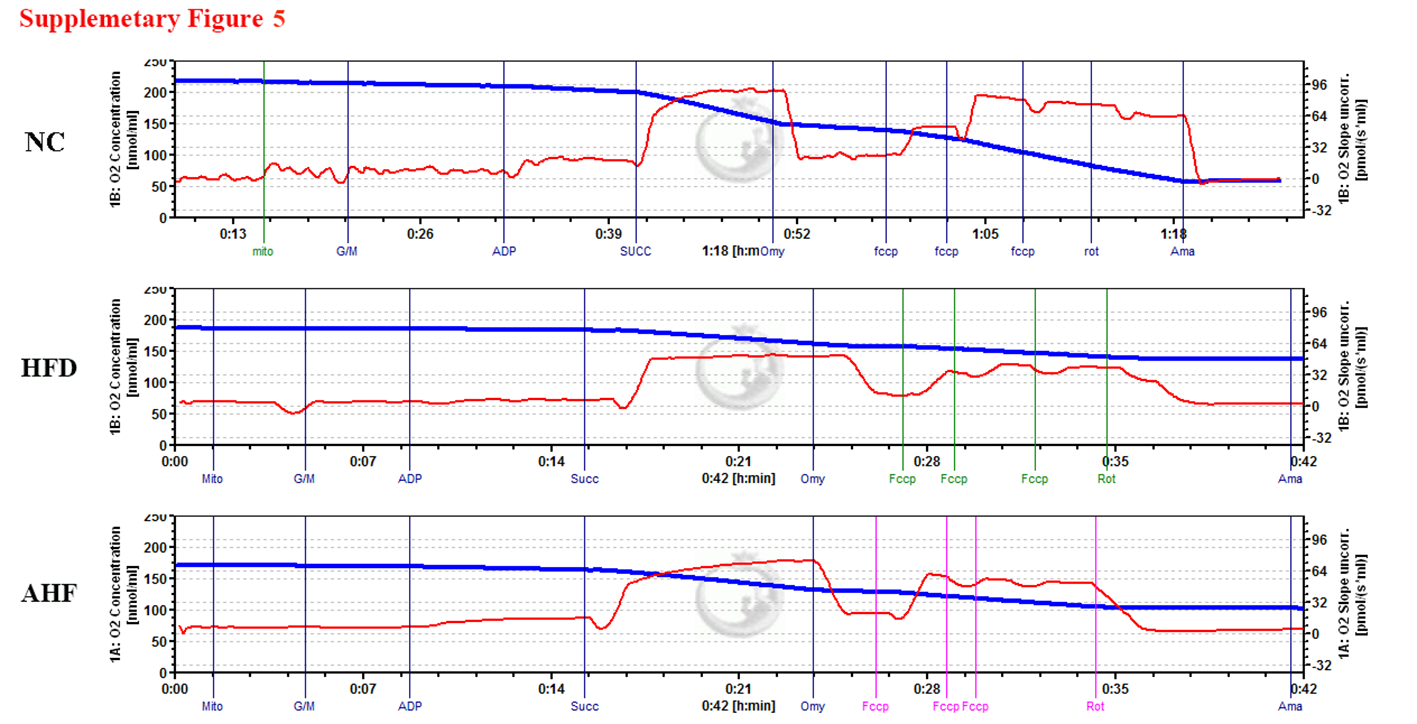

Supplement: Supplementary file 5 [file ACEL-18-e12883-s005.tif]

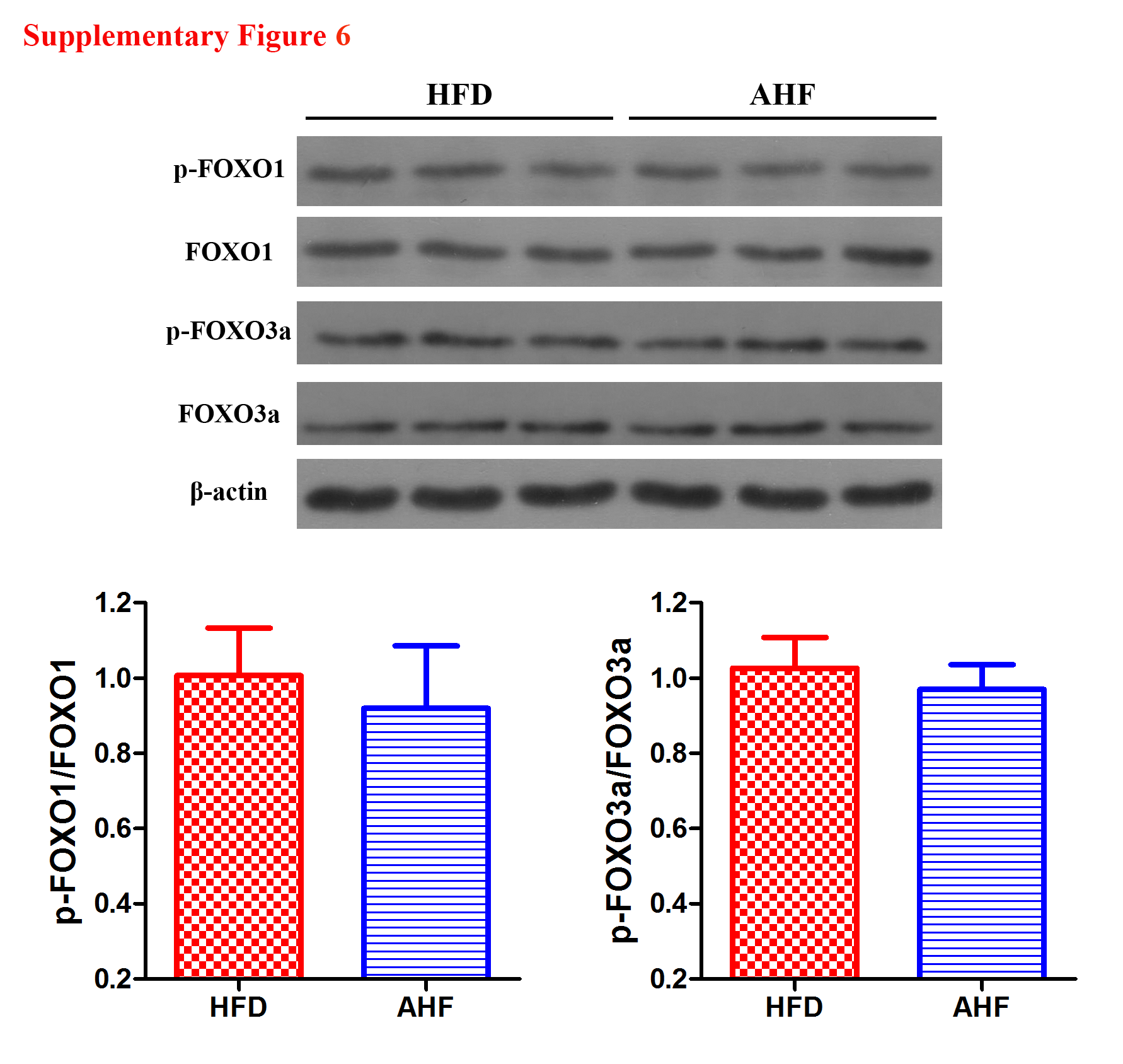

Supplement: Supplementary file 6 [file ACEL-18-e12883-s006.tif]

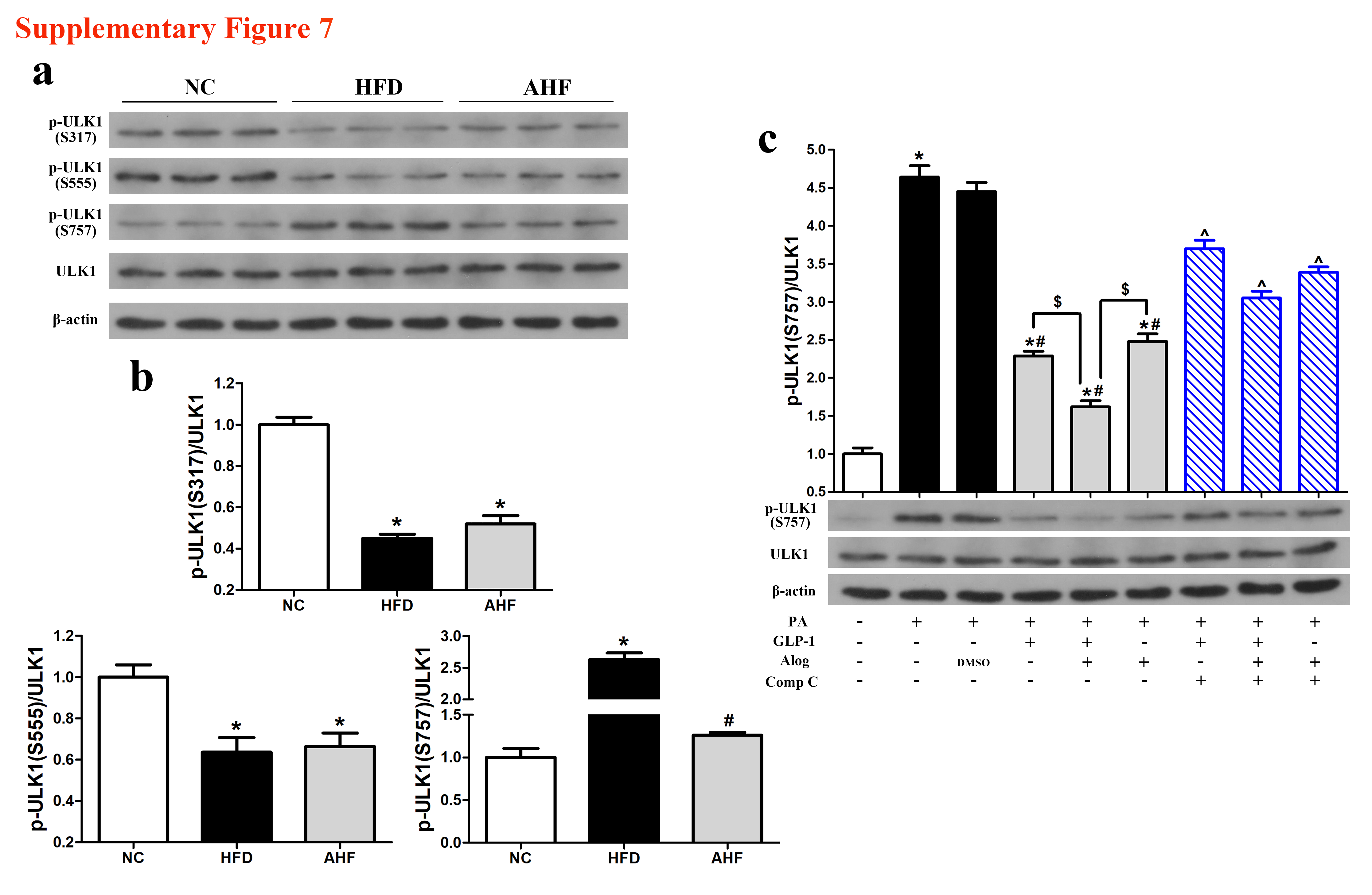

Supplement: Supplementary file 7 [file ACEL-18-e12883-s007.tif]

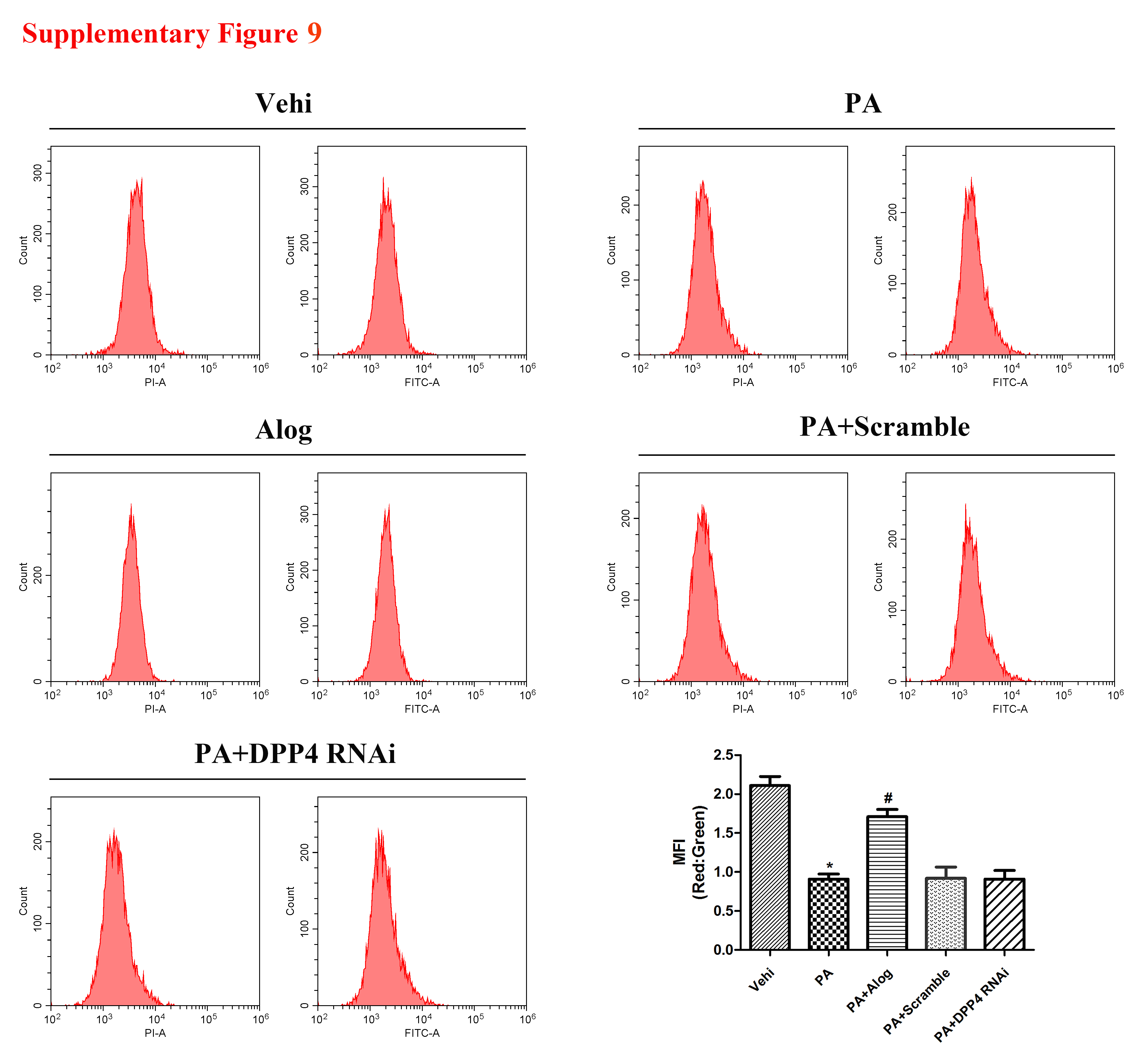

Supplement: Supplementary file 9 [file ACEL-18-e12883-s009.tif]

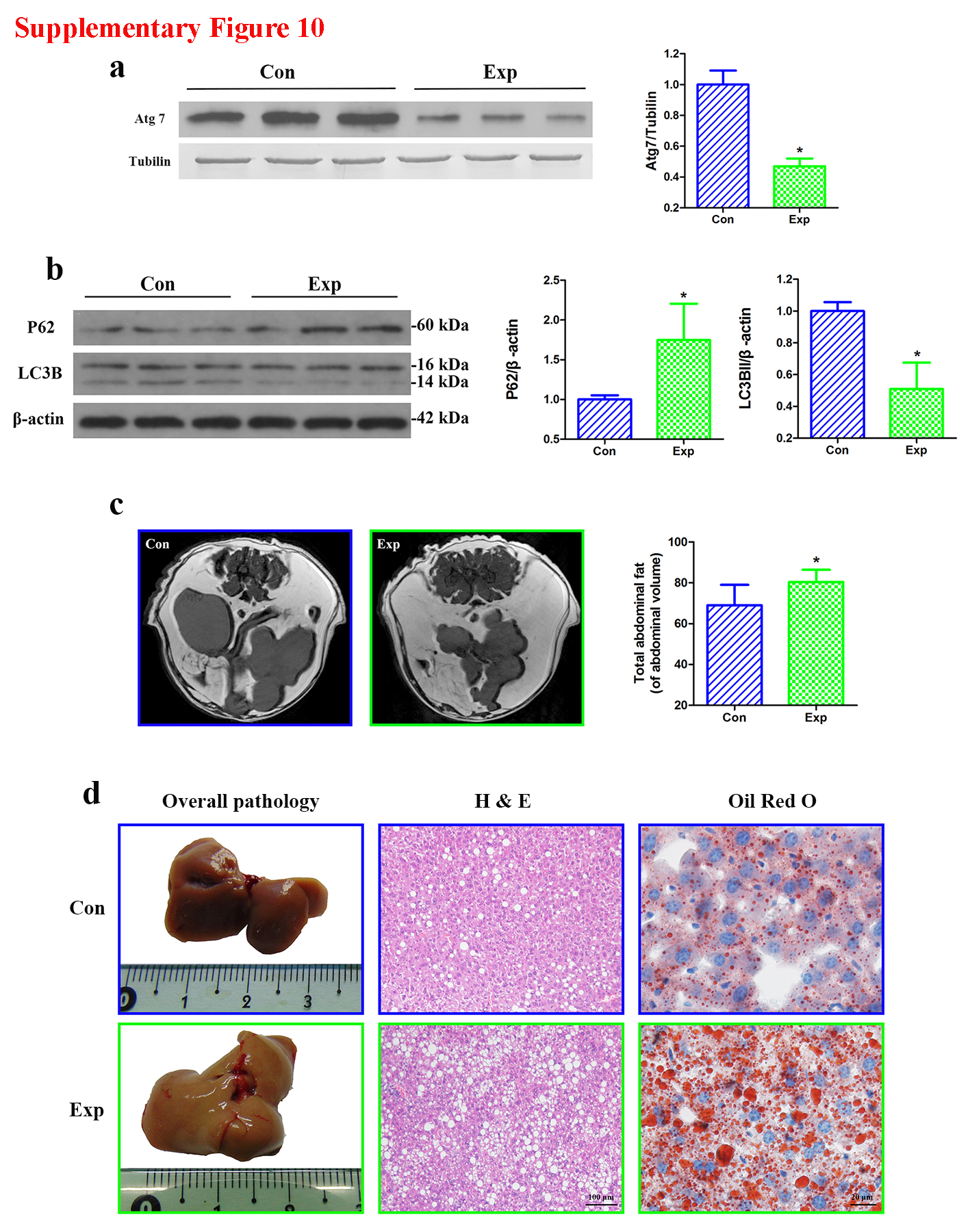

Supplement: Supplementary file 10 [file ACEL-18-e12883-s010.tif]

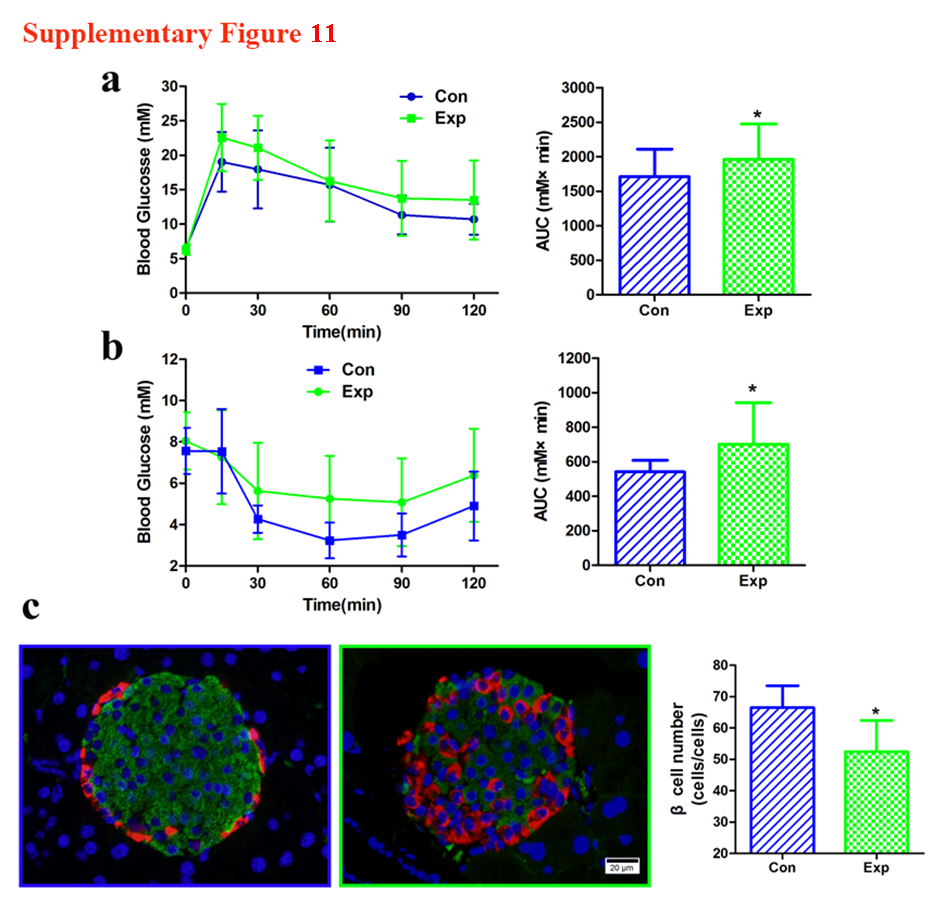

Supplement: Supplementary file 11 [file ACEL-18-e12883-s011.tif]

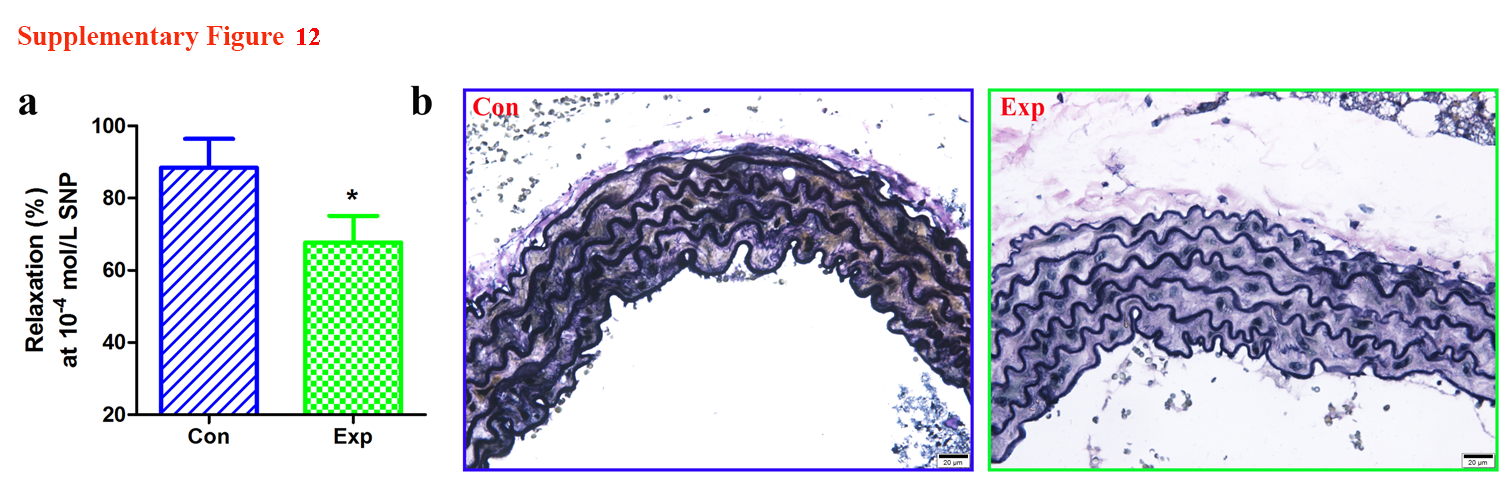

Supplement: Supplementary file 12 [file ACEL-18-e12883-s012.tif]

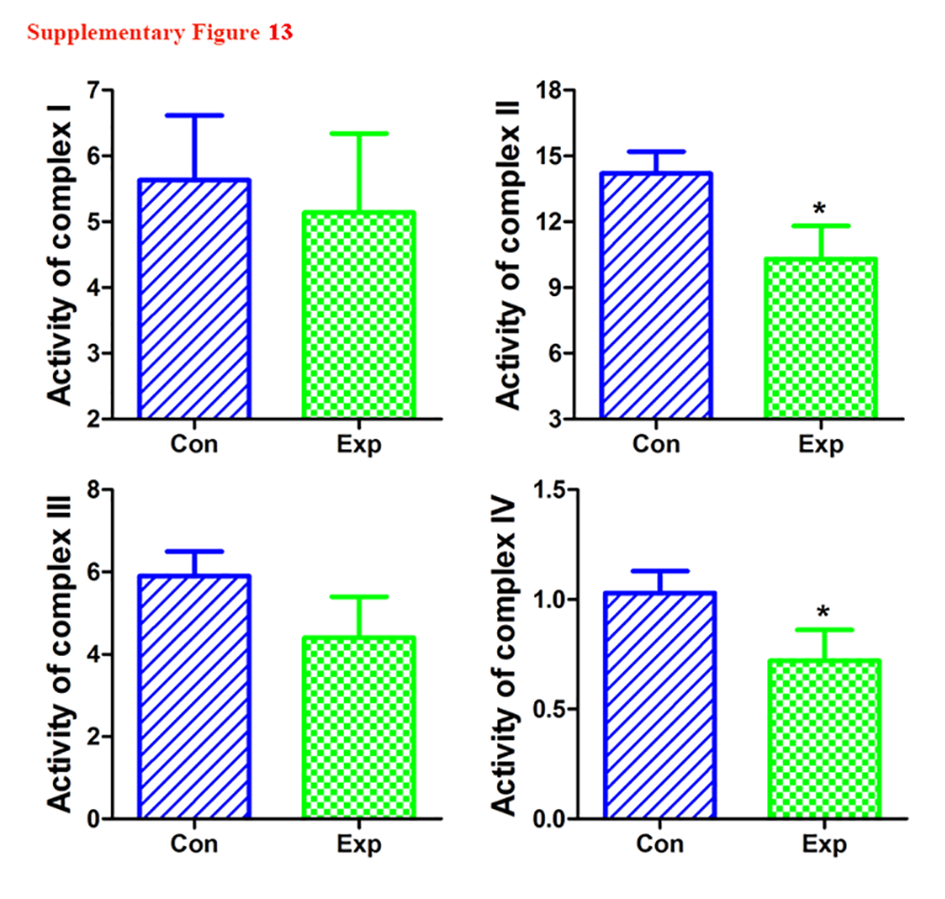

Supplement: Supplementary file 13 [file ACEL-18-e12883-s013.tif]

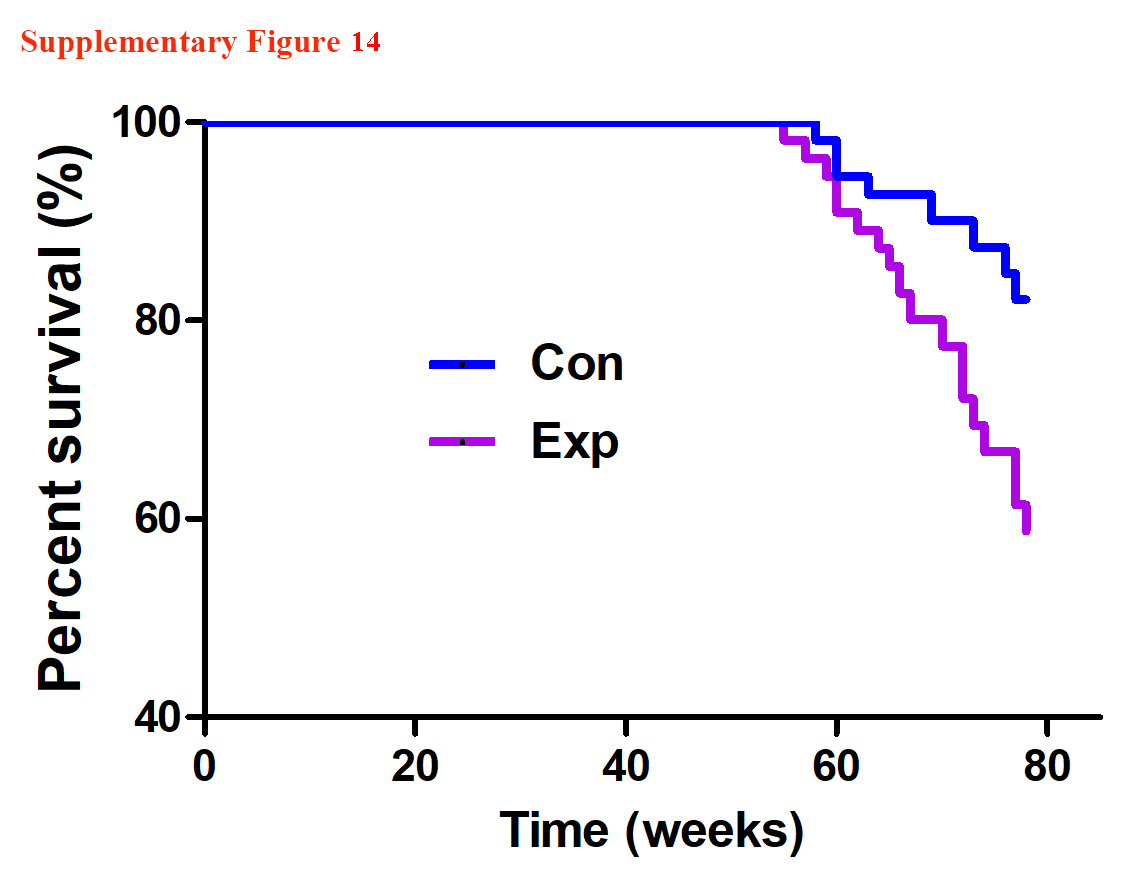

Supplement: Supplementary file 14 [file ACEL-18-e12883-s014.tif]
