# Supplementary material for: Alogliptin improves survival and health of mice on a high‐fat diet
Source: Aging Cell. 2019 Jan 15;18(2):e12883. doi: 10.1111/acel.12883 (PMC6413659; doi:10.1111/acel.12883)
Supplement: Supplementary file 17 [file ACEL-18-e12883-s017.docx]

**Alogliptin** **improves survival and health** **of mice on** **a high-fat diet**

Biao Zhu^1^, Yixiang Li^2^, Lingwei Xiang^3^, Jiajia Zhang^1^, Li Wang^1^, Bei Guo^1^, Minglu Liang^4^，Long Chen^4^, Lin Xiang^1^,Jing Dong^1^, Min Liu^1^, Wen Mei^1^, Huan Li^1^, Guangda Xiang^1*^

1. Department of Endocrinology, Wuhan General Hospital of Chinese People's Liberation Army, Wuluo Road 627, Wuhan 430070, Hubei Province, China.
2. Department of Hematology and Medical Oncology, School of Medicine, Emory University, Atlanta, GA 30322, USA.
3. Allconnect Inc., 980 Hammond Drive, Suite 1000, Atlanta, GA 30328, USA.
4. Clinical Center of Human Gene Research, Union Hospital, Tongji Medical College, Huazhong University of Science and Technology, Jiefang Ave 1277, Wuhan 430022, Hubei Province, China.

**Supplementary Methods**

**Animals and diets.** Animal procedures conformed to the National Institutes of Health Guidelines for the Use of Laboratory Animals and were approved by the Animal Ethics Committee of Wuhan General Hospital. All mice were maintained on normal chow *ad lib* for two weeks prior to the start of the experiment. The groups presented in this study were either continued feeding with normal chow (NC, *n*=55 mice), switched to high-fat chow [HFD (modified by the addition of hydrogenated coconut oil to provide 60% of calories), *n*=55 mice] or 0.03% (wt/wt) alogliptin plus HFD (AHF, *n*=55 mice). Coconut oil was chosen as previously reported^1^ to avoid high levels of dietary cholesterol associated with animal fats such as lard and because it is solid at room temperature so that a large quantity can be added to the food without much change in the consistency. This also helps to prevent the overgrowth of teeth, which may happen with high fat paste-like diets over an extended period. Alogliptin was purchased from Takeda Co., Ltd. (Osaka Plant) and mixed to homogeneity during manufacturing of the diets (Huafukang Co., Beijing). Chow was never permitted to exceed 50℃ and was kept away from light whenever possible to ensure the stability of alogliptin (the light/dark cycle in the mouse facility was not altered). All chow was stored in the dark at -20℃ and food was provided in cages for no more than one week.

**Rotarod.** Mice were tested at 15, 18, 21 and 24 months of age. At each time point the mice (*n*=15 mice per group) were given a habituation trial on day 1 when they were placed on the rotarod (ZS Dichuang, China) at a constant speed (5 rpm) and had to remain on the rotarod for 1 min. The following day, each mouse was given three trials during which the rotarod accelerated from 5 rpm to 40 rpm over a period of 5 min and time to fall was measured. The maximum trial length was 5 min and there was a 30-min rest period between each trial. Results shown are the average of three trials per mouse.

**Masticatory function.** Mice were tested at 21 months of age. We autoclaved the disposal bamboo chopsticks and cooled them at room temperature. Then, the chopsticks with the same shape and weight (*W_0_*) were selected for using. One mouse obtained an intact chopstick to nibble for 24 hours, then the residual chopsticks were weighed again (*W_X_*) and masticatory efficiency was calculated by (*W_0_*-*W_X_*)/*W_0_*.

**Heat production.** The parameter was determined using LabMaster System (TSE, Germany) in 24-month-old mice (*n*=8 mice per group). Mice were singly housed, and the first complete 12-h dark and light cycles were recorded for parameter analysis after an initial adaptation for 6 hours. Constant airflow (0.8 L/min) was drawn through the chamber and measurement in each chamber was recorded at 30-min intervals.

**IPGTT and ITT.** Blood samples were collected from 21 months old mice (*n*=10 mice per group) by tail-bleeding and analyzed as described^2^. For intraperitoneal glucose tolerance test (IPGTT), 21 months old mice (*n*=10 mice per group) were fasted for 15 hours and received an intraperitoneal (i.p.) injection of 2 g·kg^-1^ glucose. For insulin tolerance test (ITT), 21 months old mice (*n*=10 mice per group) were fasted for 5 hours and followed by an i.p. injection of 0.75 U·kg^-1^ insulin. At baseline and 15, 30, 60, 90 and 120 min after glucose or insulin administration, blood samples were obtained by tail-bleeding and glucose level was checked by a portable glucose meter (One Touch, USA), respectively. The incremental area under the curve (AUC) was calculated using the trapezoidal rule.

**Plasma DPP**-**4 enzymatic assay.** Based on a previous study^3^, after the blood sampling (*n*=10 mice per group), 10 μl of plasma was mixed with 40 μl of assay buffer containing 250 mM Tris-HCl (pH= 7.5), 0.25% (wt/vol) bovine serum albumin, and 0.125% (wt/vol) 3-[(3-Cholamidopropyl) dimethylammonio] propanesulfonic acid (CHAPS; MedChemExpress, USA) in 96-well plates. The plates were then shaken and samples were mixed with 50 μl of 1 mM Gly-Pro-pNA**·**Tos (Peptide Institute, the USA) to initiate the reactions. The samples were incubated at 30℃ on a plate shaker, and the increase in absorbance at 405 nm was monitored at both 20 and 60 min after the reaction initiation using a microtiter plate reader (Bio Tek, USA). Plasma DPP-4 activity of the vehicle-treated mice was defined as 100%.

**Blood pressure.** Blood pressure was measured as previously described^4^. Briefly, systolic and diastolic blood pressures were noninvasively measured in 24-month-old conscious animals (*n*=10 mice per group) by the tail-cuff method (Softron BP-98A, Tokyo, Japan). Blood pressure values were averaged from three consecutive measurements in steady-state conditions.

**Aortas vasodilation function assessment.** Thoracic aortas of 26-month-old mice (*n*=5 mice per group) were harvested to perform the endothelium-independent vasodilation responses as previously described^5,6^.

**PWV.** Non-invasive aortic velocities were measured in 24-month-old mice (*n*=10 mice per group) as previously described^7^. Velocities were measured with Doppler probes at the transverse aortic arch and abdominal aorta and pre-ejection times were calculated for both sites. Then, the distance between the two sites was divided by the difference in the thoracic and abdominal pre-ejection times and was presented as centimeters/second (cm/s).

**Echocardiography.** The cardiac structural and functional parameters of 24-month-old mice (*n*=10 mice per group) were detected as previously described^4,8^ by ultrasonic cardiogram using the Vevo 2100 Imaging System (VisualSonics Inc., Canada) with a 30 MHz transducer. The main parameters including left ventricle mass (LV mass), ejection fraction (EF) and fractional shortening (FS) were measured and calculated. All mice were examined by the same echocardiography to reduce the systematic error and all measurements were averaged from three consecutive cardiac cycles under stable conditions.

**Magnetic resonance imaging (MRI).** MRI experiments on 24-month-old mice (*n*=10 mice per group) were performed using a Bruker BioSpec 70/20 USR MRI with a horizontal bore 7 Tesla (Bruker BioSpec MRI GmbH, Ettlingen, Germany) as previously described^9^. T1-weighted axial images of the abdomen were obtained using a spin-echo sequence with TE=11.0 ms, TR=650.0 s, slice thickness=1.0 mm, matrix dimension=256×256, field of view=4.0×4.0 cm^2^. Measurements of fat mass were acquired using the MRI analyzer, according to the manufacturer’s instructions.

**Microcomputed tomography (micro-CT) analysis.** The left tibias dissected from 26-month-old mice (*n*=6 samples per group) were fixed in 4% paraformaldehyde at 4℃ for 48 h, then scanned and analyzed by an μCT 50 microcomputed tomography system (SCANCO MEDICAL Co.). X-ray voltage and current were set to 70 kV and 114 μA, respectively, with a resolution of 9.0 μm per pixel. To determine trabecular bone volume per tissue volume (Tb.BV/TV), trabecular number (Tb.N), trabecular separation (Tb.Sp), and trabecular thickness (Tb.Th), the region of interest (ROI) was selected at 10% of the tibia length from 0.1 mm below the growth plate for analysis. Cross-sectional images of tibias were used to perform analysis of cortical bone. The ROI selected for analysis was 10% of the tibia length in the mid-diaphysis of the tibia to determine bone mean density (BMD), periosteal perimeter (Ps.Pm), cortical thickness (Ct.Th) and endosteal perimeter (Es.Pm).

**Mitochondrial respiratory function.** Liver mitochondrial respiration was assessed using Oxygraph-2k high-resolution respirometry (Oroboros Instruments, Innsbruck, Austria) according to previously reported^4,10,11^. Basal respiration was measured when oxygen flux was stabilized. By addition of glutamate and malate to the chambers, state 2 respiration of Complex I (State 2-I) was assessed. Oxidative phosphorylation with electron flux through complex I was then quantified by the addition of ADP for assessment of State 3 respiration (State 3-I). Maximal ADP respiration with electron flux through both complex I and complex II was assessed by the addition of succinate (State 3-I+II). Then, oligomycin was added to inhibit ATP synthase and induced LEAK respiration (Leak-I+II). Subsequently, maximal capacity of the electron transport system was assessed by uncoupling with the addition of FCCP (Uncoupled-I+II). Finally, rotenone was added to determine non-coupled respiration of complex II (Uncoupled-II).

The activities of mitochondrial respiratory chain complexes I, II, III and IV in Con and Exp mice were measured by spectrophotometry using commercial kits according to the manufacturer’s instructions (GENMED, Shanghai, China).

**Serum biochemical markers.** Serum levels of glucose, TG, TC, HDL, LDL, amylase, Ala aminotransferase, Asp aminotransferase and creatinine were determined by automatic biochemical analyzer (Hitachi, Japan). FFA was measured by colorimetric assays using a commercially available kit (Jiancheng, Nanjing, China). GLP-1, insulin, IGF-1, HbA1c, adiponectin, TNF-α and IL-6 were measured using enzyme-linked immunosorbent assay (ELISA) kits (R&D, USA).

**Other parameters.** Lipid content in feces was determined as previously reportd^1^. The sequence of Atg7 siRNA is CCUGUGAGCUUGGAUCAAA. The sequence of DPP4 siRNA is GCAGGAGCUGUGAAUCCAA. Primer pairs used in RT-PCR: TNF-α forward: 5′- TGCTGGGAAGCCTAAAAGG-3′, reverse: 5′-CGAATTTTGAGAAGATGATCCTG-3′; IL-6 forward: 5′- CACATGTTCTCTGGGAAATCGTGGA-3′, reverse: 5′-TCTCTCTGAAGGACTCTGGCTTTGT-3′; GAPDH forward: 5′- TCAACAGCAACTCCCACTCTTCCA-3′, reverse: 5′-TTGTCATTGAGAGCAATG CCAGCC-3′.

**Western blotting.** Measurements of the target protein levels were performed on tissue or cell extracts using RIPA lysis buffer, and protein concentration was measured using BCA protein assay method. Equal amounts of protein for each sample were separated by SDS-PAGE and transferred onto PADF membranes. After being blocked using 5% non-fat dry milk in Tris-Buffered Saline Tween 20 (TBST) for 2 h at room temperature, the membranes were probed with the primary antibodies recognizing Sirt1 [(dilution: 1:300, Sc-15404, Santa cruz) for cell experiments; (dilution: 1:600, Ab110304, Abcam) for animal experiments], p-AMPKα (dilution: 1:1000, #2535，Cell Signaling Technologies), AMPKα (dilution: 1:1000, #5832，Cell Signaling Technologies), p-mTOR (dilution: 1:600, BS4706，Bioworld), mTOR (dilution: 1:600, BS3611，Bioworld), p-S6K (dilution: 1:1000, #9234，Cell Signaling Technologies), S6K (dilution: 1:1000, #2708，Cell Signaling Technologies), p-ULK1(S317) [dilution: 1:1000, #12753，Cell Signaling Technologies], p-ULK1(S555) [dilution: 1:1000, #5869，Cell Signaling Technologies], p-ULK1(S757) [dilution: 1:1000, #14202，Cell Signaling Technologies], ULK1 [dilution: 1:1000, #8504，Cell Signaling Technologies], P62 (dilution: 1:1000, #5114，Cell Signaling Technologies), LC3B (dilution: 1:2000, Ab192890, Abcam), p-FOXO1(dilution: 1:1000, #9461, Cell Signaling Technologies), FOXO1 (dilution: 1:1000, #9454, Cell Signaling Technologies), p-FOXO3a (dilution: 1:1000, #9466, Cell Signaling Technologies), FOXO3a (dilution: 1:1000, #2497, Cell Signaling Technologies) and Atg7 (dilution: 1:1000, #2631, Cell Signaling Technologies) overnight at 4℃, followed by incubation with horseradish peroxidase-conjugated goat anti-rabbit IgG for 1 h at room temperature. Blots were visualized using chemiluminescence reagent (Amersham Bioscience, UK). Densitometric quantification of Western data were normalized to β-actin (dilution: 1:200, BM0627, Boster Bioengineering Co., Wuhan, China) or α-Tubulin (dilution: 1:1000, #2144, Cell Signaling Technologies) serving as loading controls.

**Supplementary Figure Legends.**

**Supplementary Figure 1. DPP-4 inhibitors alogliptin and sitagliptin increase survival.** (**a**) Kaplan-Meier survival curves of ApoE^-/-^ mice (*n*=12 mice for HFD group, *n*=13 mice for LAHF group, *n*=13 mice for HAHF group). (**b**) Kaplan-Meier survival curve of sitagliptin intervention (*n*=50 mice for SHF group, *n*=55 mice for HFD group). (**c**) Total feces mass per week of each mouse. (**d**) Lipid content in feces. NC, normal chow. HFD, high-fat diet. LAHF, [low dose of alogliptin (0.01% wt/wt) plus HFD]. HAHF, [high dose of alogliptin (0.03% wt/wt) plus HFD]. AHF, 0.03% (wt/wt) alogliptin plus HFD. SHF, [0.4% (wt/wt) sitagliptin plus HFD]. Data are expressed as mean±SD. **P*<0.05 compared with NC group.

**Supplementary Figure 2. Representative echocardiograms.** Cardiac structural and functional parameters of 24-month-old mice were detected by ultrasonic cardiogram with a 30 MHz transducer. LV mass, EF and FS were measured and calculated. LV mass, left ventricle mass. EF, ejection fraction. FS, fractional shortening. NC, normal chow. HFD, high-fat diet. AHF, 0.03% (wt/wt) alogliptin plus HFD. *n*=10 mice per group.

**Supplementary Figure 3. Alogliptin intervention improves organ pathology of pancreas, adipose, liver and aorta.** (**a**) β-cell proportion. (**b**) TNF-α and IL-6 mRNA expression abdominal visceral adipose tissue. (**~~b~~ c**) Liver mass. (**~~c~~ d**) Representative aortic sections stained with hematoxylin & eosin (H&E). Scale bar, 20 μm. NC, normal chow. HFD, high-fat diet. AHF, 0.03% (wt/wt) alogliptin plus HFD. Data are expressed as mean±SD. **P*<0.05 compared with NC group, **^#^***P*<0.05 compared with HFD group.

**Supplementary Figure 4.** **Alogliptin intervention improves organ pathology of tibia and aorta.** (**a**, **b**) Alogliptin intervention attenuates bone loss. Representative images of micro-CT (**a**) and quantitative analysis of bone microarchitectures (**b**). (**c**) Representative aortic sections stained with van Gieson (VG). Scale bar, 20 μm. Tb.BV/TV, trabecular relative volume; Tb.N, trabecular number; Tb.Sp, trabecular separation; Tb.Th, trabecular thickness; BMD, bone mean density; Ps.Pm, periosteal perimeter; Ct.Th, cortical thickness; Es.Pm, endosteal perimeter. NC, normal chow. HFD, high-fat diet. AHF, 0.03% (wt/wt) alogliptin plus HFD. Data are expressed as mean±SD. **P*<0.05 compared with NC, **^#^***P*<0.05 compared with HFD.

**Supplementary Figure 5. Representative images of oxygen consumptions.** Liver mitochondria from 26 months old mice were harvested. Then, substrates, uncoupler and inhibitors were applied to analyze respiratory function using Oxygraph-2k high-resolution respirometry. NC, normal chow. HFD, high-fat diet. AHF, 0.03% (wt/wt) alogliptin plus HFD. *n*=6 mice per group.

**Supplementary Figure 6. FOXO signaling** **did not involve in promoting health and longevity.** Western blots of total liver lysates for p-FOXO1, FOXO1, p-FOXO3a and FOXO3a. β-Actin was used as a loading control. Densitometric quantification for the expression of the proteins p-FOXO1 and p-FOXO3a was performed using ImageJ. HFD, high-fat diet. AHF, 0.03% (wt/wt) alogliptin plus HFD. *n*=3 samples per group.

**Supplementary Figure 7. AMPK-ULK1 pathway did not involve in autophagy activation by alogliptin intervention.** (**a**) Western blots of total liver lysates for phosphorylated (p)-ULK1(S317), p-ULK1(S555) and p-ULK1(S757), β-Actin was used as a loading control. (**b**) Densitometric quantification of (**a**). (**c**) Primary hepatocytes were pretreated with PA (0.4 mM) for 24 h, and then added Compound C (20 µM) for 30 min, following added Alog (50 µM) or DMSO (solvent of Alog) for 30 min, finally added GLP-1 (100 nM) where indicated. After incubation without changing culture medium for another 24 h, cells were harvested for Western blotting. Representative immunoblots and densitometric quantification for the expression of p-ULK1(S757). NC, normal chow. HFD, high-fat diet. AHF, 0.03% (wt/wt) alogliptin plus HFD. PA, palmitic acid. Alog, Alogliptin. Com C, Compound C. **P*<0.05 compared with NC or vehicle group, **^#^***P*<0.05 compared with HFD or PA group, ^*P*<0.05 compared with corresponding groups without inhibitors, $ *P*<0.05. Data are expressed as mean±SD. Each experiment repeated 3 times.

**Supplementary Figure 8. Alogliptin administration stimulates autophagy *in vitro*.** (**a-f**) L-02 cells were pretreated with PA (0.4 mM) for 24 h, and then added inhibitors [Compoud C (20 µM), RAPA (1µM) or CQ (10 µM)] for 30 min, following added Alog (50 µM) or DMSO (solvent of Alog) for 30 min, finally added GLP-1 (100 nM) where indicated. After incubation without changing culture medium for another 24 h, L-02 cells were harvested for Western blotting. Representative immunoblots and densitometric quantification for the expression of proteins Sirt1 (**a**), phosphorylated (p)-AMPK (**b**), p-mTOR (**c**), p-S6K (**d**), p62 (**e**) and LC3B (LC3BII, 14 kDa) (**f**). **P*<0.05 compared with the vehicle group, **^#^***P*<0.05 compared with PA group, ^*P*<0.05 compared with corresponding groups without inhibitors, $ *P*<0.05. (**g-h**) L-02 cells were pretreated with PA (0.4 mM) for 24 h, and then added Alog (50 µM) for 30 min, following added GLP-1 (100 nM), if indicated. Then the cells were incubated without changing culture medium for another 24 h. Adenoviruses encoding mRFP-GFP-LC3 were transfected 48 h prior to harvest. (**g**) Representative ﬂuorescence micrographs. Scale bar, 10 μm. (**h**) Quantification of (**g**), **P*<0.05. PA, palmitic acid. RAPA, rapamycin. CQ, chloroquine. (**i-j**) Primary hepatocytes were pretreated with PA (0.4 mM) for 24 h, and then added Bafilomycin (100 nM), Alog (50 µM) for 30 min, following added GLP-1 (100 nM), if indicated. Then the cells were incubated without changing culture medium for another 24 h. cell lysates were quantified by Western blot analysis. The differences in the amount of LC3B-II (14kDa) between groups in the presence and absence of bafilomycin represent the amount of LC3B‑II that is delivered to lysosomes for degradation. **P*<0.05 compared with the vehicle group, **^#^***P*<0.05 compared with PA group. Data are expressed as mean±SD. Each cell experiment repeated 5 times.

**Supplementary Figure 9. Effects of DPP-4 inhibition on autophagy flux.** Primary hepatocytes were pretreated with PA (0.4 mM) for 24 h, following added Alog (50 µM), DPP4 siRNA (20 µM) or scramble, if indicated. Then the cells were incubated for another 24 h without changing culture medium and assessed by flow cytometry (FITC, Red; PI, Green). Adenoviruses encoding mRFP-GFP-LC3 were transfected 48 h prior to harvest. PA, palmitic acid. Alog, alogliptin. MFI, mean fluorescence intensity. **P*<0.05 compared with the vehicle group, **^#^***P*<0.05 compared with PA group.

**Supplementary Figure 10. Autophagy is required for decreasing adipose deposition.** (**a**) Immunoblots and densitometric quantification for the expression of Atg7. (**b**) Immunoblots and densitometric quantification for the expression of p62 and LC3B (LC3BII, 14 kDa). (**c**) T1-weighted spin-echo images and analysis of fat mass distribution. (**d**) Liver pathology assessed by organ size, hematoxylin-eosin (H&E) staining (scale bar, 100 μm) and oil red O staining (scale bar, 20 μm). Con, control group. Exp, experimental group. Data are expressed as mean±SD. **P*<0.05 compared with Con group.

**Supplementary Figure** **11. Autophagy is required for elevating glucose disposal ability.** (**a**) Plasma levels of glucose after intraperitoneal glucose tolerance test (IPGTT) and area under each curve. (**b**) Plasma levels of glucose after insulin tolerance test (ITT) and area under each curve. (**c**) Representative images of pancreatic sections double-stained for insulin (green) and glucagon (red), the nuclei were stained with DAPI (blue). Scale bar, 20 μm. Con, control group. Exp, experimental group. Data are expressed as mean±SD. **P*<0.05 compared with Con group.

**Supplementary Figure 12. Autophagy is required for improving health of aortas.** (**a**) Endothelium -independent vasodilation function of aortas in response to sodium nitroprusside (SNP). (**b**) Representative aortic sections stained with van Gieson (VG). Scale bar, 20 μm. Con, control group. Exp, experimental group. Data are expressed as mean±SD. **P*<0.05 compared with Con group.

**Supplementary Figure 13. Autophagy is required for elevating complex activity of electron transport chain.** Activities of mitochondrial respiratory chain complexes I, II, III and IV in the liver of Con and Exp mice were measured by spectrophotometry. Con, control. Exp, experimental. Data are expressed as mean±SD. **P*<0.05 compared with Con group.

**Supplementary Figure 14. Autophagy is required for extending longevity.** C57BL/6J mice were randomly assigned to Con group (*n*=55 mice) and Exp group (*n*=55 mice). All mice were fed with AHF. Exp mice were injected with adenovirus-encoding Atg7 siRNA and Con mice were injected with control siRNA once every three weeks. Survival curves were analyzed by Kaplan-Meier method. Con, control group. Exp, experimental group. AHF, 0.03% (wt/wt) alogliptin plus HFD.

**References:**

1. Baur, J.A.*, et al.*. Resveratrol improves health and survival of mice on a high-calorie diet. *NATURE* **444**, 337-342 (2006).

2. Li, H.*, et al.*. GDF11 Attenuates Development of Type 2 Diabetes via Improvement of Islet beta-Cell Function and Survival. *DIABETES* **66**, 1914-1927 (2017).

3. Moritoh, Y., Takeuchi, K., Asakawa, T., Kataoka, O. & Odaka, H. Chronic administration of alogliptin, a novel, potent, and highly selective dipeptidyl peptidase-4 inhibitor, improves glycemic control and beta-cell function in obese diabetic ob/ob mice. *EUR J PHARMACOL* **588**, 325-332 (2008).

4. Eisenberg, T.*, et al.*. Cardioprotection and lifespan extension by the natural polyamine spermidine. *NAT MED* **22**, 1428-1438 (2016).

5. Mei, W.*, et al.*. GDF11 Protects against Endothelial Injury and Reduces Atherosclerotic Lesion Formation in Apolipoprotein E-Null Mice. *MOL THER* **24**, 1926-1938 (2016).

6. Lu, J.*, et al.*. Irisin protects against endothelial injury and ameliorates atherosclerosis in apolipoprotein E-Null diabetic mice. *ATHEROSCLEROSIS* **243**, 438-448 (2015).

7. Fleenor, B.S.*, et al.*. Superoxide signaling in perivascular adipose tissue promotes age-related artery stiffness. *AGING CELL* **13**, 576-578 (2014).

8. Lu, Q.*, et al.*. Angiogenic Factor AGGF1 Activates Autophagy with an Essential Role in Therapeutic Angiogenesis for Heart Disease. *PLOS BIOL* **14**, e1002529 (2016).

9. Shah, Z.*, et al.*. Long-term dipeptidyl-peptidase 4 inhibition reduces atherosclerosis and inflammation via effects on monocyte recruitment and chemotaxis. *CIRCULATION* **124**, 2338-2349 (2011).

10. Aroor, A.R.*, et al.*. Dipeptidyl peptidase-4 inhibition ameliorates Western diet-induced hepatic steatosis and insulin resistance through hepatic lipid remodeling and modulation of hepatic mitochondrial function. *DIABETES* **64**, 1988-2001 (2015).

11. Li, P.*, et al.*. Mitochondrial respiratory dysfunctions of blood mononuclear cells link with cardiac disturbance in patients with early-stage heart failure. *Sci Rep* **5**, 10229 (2015).
